# Reliability analysis of inverted exponentiated Rayleigh parameters via progressive hybrid censoring data with applications in medical data

**DOI:** 10.1371/journal.pone.0336169

**Published:** 2025-12-30

**Authors:** Said G. Nassr, O.E. Abo-Kasem, Rana H. Khashab, Etaf Alshawarbeh, Shokrya S. Alshqaq, Neema M. Elharoun

**Affiliations:** 1 Department of Statistics and Insurance, Faculty of Commerce, Arish University, Al-Arish, Egypt; 2 Department of Statistics, Faculty of Commerce, Zagazig University, Zagazig, Egypt; 3 Mathematics Department, Faculty of Sciences, Umm Al-Qura University, Makkah, Saudi Arabia; 4 Department of Mathematics, College of Science, University of Ha’il, Ha’il, Saudi Arabia; 5 Department of Mathematics, College of Science, Jazan University, Jazan, Saudi Arabia; Universidad Rey Juan Carlos, SPAIN

## Abstract

This paper examines the estimation of model parameters, reliability, and hazard rate functions of the inverted exponentiated Rayleigh distribution under progressive hybrid Type-I censoring. Three estimation methodologies, maximum likelihood, maximum product of spacing, and Bayesian approaches, are explored. The classical perspective employs maximum likelihood and maximum product of spacing approaches for estimating unknown parameters, reliability, hazard rate functions, and computing approximate confidence intervals. Bayesian estimation is formulated using the squared-error and LINEX loss functions, predicated on independent gamma priors. Owing to the complex nature of the joint posterior distribution, Bayes estimates are evaluated by generating samples from the whole conditional distributions via Markov Chain Monte Carlo methods. The highest posterior density credible intervals are also established for each unknown parameter, reliability, and hazard rate functions. The efficacy of the proposed strategies is evaluated through a simulated study. To assess the efficacy of the estimation techniques, a comprehensive simulation study is conducted, encompassing various scenarios with diverse sample sizes and progressive censoring schemes. Furthermore, the practical applicability of the proposed methods is demonstrated through the analysis of real-world datasets taken from the medical field. This data represents the relief time (in hours) of arthritis patients receiving a fixed dose of this drug. Numerical investigations reveal that Bayes estimates employing the LINEX loss function exhibit superior performance compared to other estimation methods, underscoring their preference due to heightened accuracy and robustness.

## 1 Introduction

Researchers frequently do reliability and life testing tests in diverse practical investigations, including survival analysis, clinical trials, and industrial or mechanical applications. These studies collect observable data to infer unknown values of interest, like failure rates, quantiles, and reliability characteristics. The efficacy of these conclusions depends on the information included in the observed data. Nonetheless, the results observed in these tests are censored in numerous instances. censoring transpires when comprehensive information about the event of interest is inaccessible. Data censoring occurs for various reasons, including the predetermined duration of trials or the non-occurrence of specific events at the time of analysis. Numerous censoring approaches have been developed in literature to analyze suppressed data appropriately. Type I and Type II censoring systems are the most employed procedures. Type I censoring occurs when a life testing experiment is performed for a specified duration, referred to as *T*. In the experiment, a specific quantity of items, referred to as *n*, undergoes examination. No additional observations are documented for any object once the experiment reaches the predetermined time point *T*. Type I censoring permits researchers to examine the recorded failure times of items until time *T*. This allows them to deduce the pertinent unknown values and estimate failure probabilities, quantiles, reliability characteristics, and other associated metrics from the available censored data.

Type II censoring continues the experiment until *m* failures (less than or equal to *n*) are observed. However, type I censoring may conclude the experiment before gathering enough failure observations, while type II censoring may prolong the experiment. Type I hybrid censoring, suggested by Epstein [1], addresses these restrictions by combining type I and type II censoring. This hybrid technique balances managing experimental time and collecting enough failure observations. In type I hybrid censoring, the experiment ends at a random time *T*_0_, which is the minimum of the *m*-th failure time (*X*_*m*_) and a pre-specified time *T*. The experiment stops when *m* failures are seen or *T* is reached, whichever comes first. However, Childs et al. [2] proposed type II hybrid censoring, where the experiment ceases when either (*m*) failure times are observed or *T* is reached. Here, $T_{0}=max(X_{m},T)$ is the experiment’s end time. These hybrid censoring schemes randomly generate failure observations. Existing censoring schemes, including type I and type II hybrid censoring, do not allow live units to be removed from a life test at any point other than the final termination point.

In certain experimental scenarios, there arises a necessity to remove units throughout the duration of the test. Balakrishnan and Aggarwala [3] have highlighted the benefits of such flexibility in specific contexts. Allowing for unit removal at points other than the final termination enhances experimental efficiency while ensuring observation of extreme lifetimes. This flexibility proves advantageous when some surviving units can be repurposed or when practical constraints necessitate early removal, such as accidents or loss of contact with subjects. Progressive censoring emerges as a viable solution in situations where such unpredictability occurs. It permits the removal of surviving units before the test’s conclusion, thus accommodating unforeseen events during the experiment. Kundu and Joarder [4] and Childs et al. [5] have integrated type I hybrid censoring and progressive censoring into the progressive hybrid type I censoring scheme (PHT-ICS). This scheme combines the merits of both censoring approaches, aiming to optimize experimental design and data collection processes.

**Fig 1:**
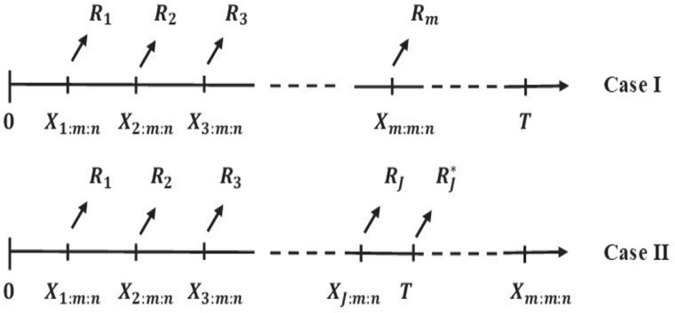
Schematic representation of PHT-ICS.

The PHT-ICS censoring system is $\left(R_{1},R_{2},...,R_{m}\right)$, where *m* is the predefined number of failures and *n* is the total number of units initially placed on the life test. This scheme’s main feature is that some surviving units can be removed before the trial ends. Take a life test with *n* test units and a progressive censoring method $\left(R_{1},R_{2},...,R_{m}\right)$ before the experiment. A predetermined time point *T* is also set in advance. Upon the first failure *X*_1:*m*:*n*_, *R*_1_ surviving units are randomly picked and removed from the experiment. During the second failure *X*_2:*m*:*n*_, *R*_2_ units are removed from the remaining $\left(n\;-\;R_{1}\;-\;2\right)$ units. The procedure repeats, removing *R*_*i*_ units from the pool of $\left(n\;-\;R_{1}\;-\;R_{2}\;-\;\ldots \;-\;R_{i-1}\;-\;i\right)$ surviving units at each failure. The experiment continues until reaching the termination point T^*^, which is the minimum of the predefined time *T* and the time of the *m*-th failure *X*_*m*:*m*:*n*_. If the *m*-th failure happens before the predetermined time *T*, the observed failures in the life test are $X_{1:m:n},X_{2:m:n},\ldots ,X_{m:m:n}$. When the *m*-th failure occurs, the test is ended and the remaining units are eliminated. The number of units to be deleted is *R*_*m*_, computed as $R_{m}=n\;-\;m\;-\;\sum_{i=1}^{m-1}R_{i}$. However, if the time *T* is reached before the *m*-th failure (*X*_*m*:*m*:*n*_>*T*), the observed life test failures are $X_{1:m:n},...,X_{j:m:n}$ (where *j* < *m*). At time *T*, the test ends and the remaining units are eliminated. At this point, the number of units to be deleted is estimated as $R_{j}^{*}=n-j-\sum_{i=1}^{j}R_{i}$ (see Fig 1).

Significant contributions to statistical inference regarding PHT-ICS have been made by various authors, including references [Kayal et al. [6], Park et al. [7]. For a comprehensive understanding of this topic, valuable resources can be found in the monograph by Balakrishnan and Cramer [8] and the review artile by Balakrishnan and Kundu [9]. These references provide up-to-date accounts and insights into the PHT-I censoring scheme. Additionally, for further inferential results and related information on this scheme and other schemes of importance in life testing, references such as [Hemmati and Khorram [10], Lin and Huang [11], Tomer and Panwar [12], Abu El Azm et al. [13], Nassr et al. [14], Yousef et al. [15], Nassar et al. [16], and Nassr et al. [17] are recommended. These sources offer additional useful findings and findings that can enhance one’s understanding of the PHT-ICS.

PHT-ICS offers several advantages over traditional censoring schemes by combining the fixed-time stopping rule of Type-I censoring, the failure-based stopping of Type-II, and the flexibility of progressive censoring. Its key advantages include:

- **Flexibility:** It allows the removal of surviving units at intermediate stages, improving efficiency and reducing experimental costs.
- **Realism:** It closely reflects real-world testing environments where data may be censored due to time constraints, early failures, or resource limitations.
- **Improved estimation:** It often yields more informative data than fixed censoring, especially in small or expensive samples.

Inverted distributions are increasingly used in statistical modeling due to their flexibility in capturing a wide range of hazard rate behaviors, including decreasing, increasing, and non-monotonic patterns. These distributions are particularly useful when the event of interest becomes less likely over a common scenario in many real-world systems. In engineering, for example, inverted distributions are applied in reliability analysis of electronic components where failure rates often decrease after initial use (known as the "infant mortality" phase). In the medical field, they are employed in modeling patient survival times under specific treatments, such as the time until recurrence of cancer after therapy, where the risk of relapse may decline over time. Economically, they serve to analyze the duration of unemployment spells, especially in situations where the probability of finding a job increases the longer an individual remains unemployed. These distributions also accommodate censored and incomplete data, making them well-suited for practical applications where full observation is not always possible. Their mathematical flexibility and practical relevance have positioned them as essential tools in modern survival and reliability analysis. Numerous authors have noted the significance and usefulness of inverted distributions in the fields of engineering, medicine, and economics [see, for example, Calabria and Pulcini [18], Sharma et al. [19], Abd AL-Fattah et al. [20], Tahir et al. [21], Hassan and Nassr [22], Hassan and Nassr [23], Nassr et al. [24], Al Mutairi et al. [25], El-Saeed et al. [26], Nassr et al. [27], Abushal et al. [28], Al Mutairi et al [29] and Alotaibi et al. [30].

The inverted exponentiated Rayleigh (IER) distribution, introduced by Ghitany et al. [31], has recently gained notable attention in the literature for its ability to model a wide variety of hazard rate behaviors, especially decreasing and non-monotonic patterns. This makes it highly suitable for modeling lifetime data in reliability engineering, medical survival studies, and economic duration analysis. He suggested IER distribution with the probability density function (PDF), cumulative distribution function (CDF), reliability function (RF), hazard rate function (HRF), and cumulative hazard function (CH) as follows:

$$f(x;\theta ,\lambda )=2\;\theta \;\lambda \;x^{-3}\;{\left[1-\exp(-\lambda x^{-2})\right]}^{\theta -1}\;\exp(-\lambda x^{-2});\;x>0,\;\theta ,\;\lambda >0,$$ (1)

$$F(x;\theta ,\lambda )=1-{\left[1-\exp(-\lambda x^{-2})\right]}^{\theta };\;x>0,\;\theta ,\;\lambda >0,$$ (2)

$$R(x;\theta ,\lambda )={\left[1-\exp(-\lambda x^{-2})\right]}^{\theta };\;x>0,\;\theta ,\;\lambda >0,$$ (3)

$$H(x;\theta ,\lambda )=2\;\theta \;\lambda \;x^{-3}\;\exp\left(-\lambda x^{-2}\right){\left[1-\exp\left(-\lambda x^{-2}\right)\right]}^{-1}\;;\;x>0,\;\theta ,\;\lambda >0,$$ (4)

and

$$CH(x;\theta ,\lambda )=-\log\left({\left[1-\exp(-\lambda x^{-2})\right]}^{\theta }\right)=-\theta \log\left(1-\exp(-\lambda x^{-2})\right);\;x>0,\;\theta ,\lambda >0.$$ (5)

where; θ and λ are the shape and the scale parameters respectively.

**Fig 2:**
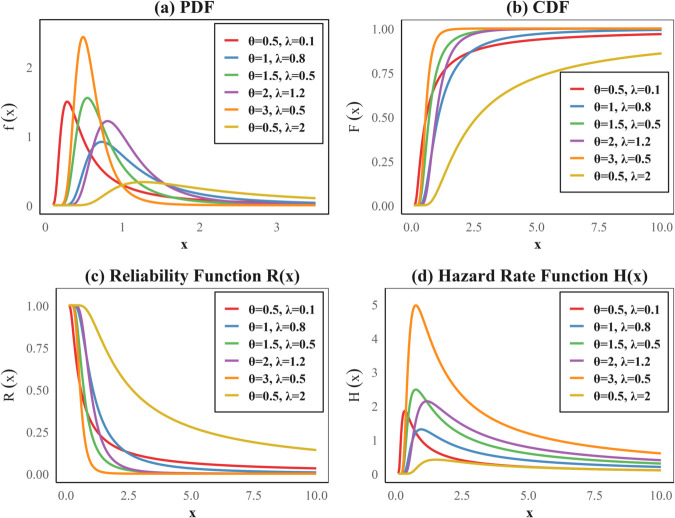
IER model functions: PDF, CDF, reliability function, and hazard for varying θ and λ. Panels (a)–(d) correspond to these functions, respectively.

As shown in Fig 2, the IER model functions demonstrate varying behavior according to parameter values, with panel (a) presenting the PDF, panel (b) the CDF, panel (c) the reliability function *R*(*x*), and panel (d) the hazard function *H*(*x*), illustrating the statistical properties of the model.

The choice of the IER distribution in this study is driven by its high flexibility in modeling diverse hazard rate behaviors, including increasing, decreasing, and bathtub-shaped functions. These characteristics make it a powerful alternative in reliability and survival analysis, particularly under censoring schemes. Moreover, recent comparative studies have demonstrated its superior fitting ability over classical models when applied to complex lifetime data. Many scholars have studied the IER distribution’s theories and applications. Rastogi and Tripathi [32] used maximum likelihood and Bayesian estimation for point and interval estimations under censored data for this distribution. Kayal et al. [33] also studied hybrid censoring scenarios for point and interval predictions for one and two samples, respectively, for the IER distribution. Kohansal [34] used Gibbs sampling to build the Bayesian estimator for the IER distribution’s stress-strength reliability. Gao et al. [35] introduced important inference methods for estimating the two unknown parameters of the IER distribution using progressive censored data. Fan and Gui [36] used maximum likelihood and Bayesian methods to estimate unknown parameters of the IER distribution under joint progressively type-II censoring. Maurya et al. [37] discuss the estimation and prediction methods for an IER distribution under progressive first-failure censoring, using maximum likelihood and Bayesian approaches. Panahi and Moradi [38] examined maximum likelihood and Bayesian approaches for the IER distribution based on an adaptive progressive hybrid censoring scheme. Anwar et al. [39] examines stress-strength reliability estimation for the IER distribution using unified progressive hybrid censoring, employing maximum likelihood estimation via the stochastic EM algorithm and Bayesian approaches with Gibbs/Metropolis-Hastings sampling. Salem et al. [40] examine point and interval estimates for IER distribution under a general progressive Type-II censoring scheme, employing both maximum likelihood and Bayesian approaches. Recent studies have also explored the inverted exponentiated Rayleigh (IER) distribution under various censoring schemes.

For instance, Wang et al. [41] investigated estimation and prediction under a *modified* progressive hybrid censoring scheme, whereas our study adopts the standard progressive hybrid Type-I censoring scheme (PHT-ICS). Hashem et al. [42] applied empirical Bayes estimation to progressively hybrid censored medical data, but assumed the scale parameter to be known. In contrast, we jointly estimate both shape and scale parameters using full likelihood-based and Bayesian methods (under squared-error and LINEX loss functions), with a comprehensive assessment that includes simulation, model diagnostics, and real-data validation. These distinctions broaden the applicability and rigor of the proposed framework.

The remaining sections of the paper are structured in the following manner: The maximum likelihood estimators (MLEs) and asymptotic confidence intervals (ACIs) for the unknown parameters θ,λ,R(x), and *H*(*x*) are examined in Sect 2. The point estimators and ACIs using the maximum product of spacing estimation (MPSE) are derived in Sect 3. Sect 4 discusses the application of Bayesian estimating. Sect 5 contains a detailed presentation of the simulation technique and its findings. Sect 6 presents the study of an actual data set that pertains to the field of medicine. Sect 7 contains the concluding remarks.

## 2 Maximum likelihood estimation

Suppose a test employing the PHT-ICS involves *n* units sampled from the IER distribution. The observed data may fall into one of two possible scenarios regarding the censoring scheme:

Case I: $\left\{X_{1:m:n},\;X_{2:m:n},\;\ldots ,\;X_{m:m:n}\right\}$, if *X*_*m*:*m*:*n*_<*T*,

Case II: $\left\{X_{1:m:n},\;X_{2:m:n},\;\ldots ,\;X_{d:m:n}\right\}$, if $X_{d:m:n}<T<X_{d+1:m:n}$.

Then, the likelihood function with PHT-ICS is


$$L\left(\theta ,\lambda |x\right)\propto \prod_{i=1}^{r}f\left(x_{i};\theta ,\lambda \right){\left[R\left(x_{i};\theta ,\lambda \right)\right]}^{R_{i}}\;{\left[R\left(C;\theta ,\lambda \right)\right]}^{R_{T}},$$

where; *r* = *m*, $C=x_{\left(m\right)}$, *R*_*T*_ = 0 in case I, while in case II, *r* = *d*, *C* = *T*, $R_{T}=\left(n-d-\sum_{i=1}^{d}R_{i}\right)$ and $x_{i}=x_{\left(i\right)}$, based on the observed data, the likelihood function can be expressed as:

$$L\left(\theta ,\lambda |x\right)\propto \theta^{r}\lambda^{r}\;\;e^{-\lambda \sum_{i=1}^{r}x_{i}^{-2}}\left\{{\left(1-e^{-\lambda x_{i}^{-2}}\right)}^{\theta R_{T}}\right\}\left\{\prod_{i=1}^{r}\left[{\left(1-e^{-\lambda x_{i}^{-2}}\right)}^{\theta \left(1+R_{i}\right)-1}\right]\right\}.$$ (6)

Then, the log likelihood function, denoted by lnL=L(θ,λ|x) can be written as


$$lnL\;=rln\theta \;+rln\lambda \;-\lambda \sum_{i=1}^{r}x_{i}^{-2}+\sum_{i=1}^{r}\left[\theta \left(1+R_{i}\right)-1\right]ln\left(1-e^{-\lambda x_{i}^{-2}}\right)\;+\theta R_{T}ln\left(1-e^{-\lambda C^{-2}}\right)\;$$

The first partial derivative for the unknown parameters as follow:-

$$\frac{\partial lnL\;}{\partial \theta }=\frac{r}{\theta }+\sum_{i=1}^{r}\left(1+R_{i}\right)ln\left(1-e^{-\lambda x_{i}^{-2}}\right)\;+R_{T}ln\left(1-e^{-\lambda C^{-2}}\right)\;,$$ (7)

$$\frac{\partial lnL\;}{\partial \lambda }=\frac{r}{\lambda }-\sum_{i=1}^{r}x_{i}^{-2}+\sum_{i=1}^{r}\left[\theta \left(1+R_{i}\right)-1\right]\;x_{i}^{-2}\;\;e^{-\lambda x_{i}^{-2}}{\left(1-e^{-\lambda x_{i}^{-2}}\right)}^{-1}$$

$$+\theta R_{T}\;\;C^{-2}\;\;e^{-\lambda C^{-2}}{\left(1-e^{-\lambda C^{-2}}\right)}^{-1}.$$ (8)

From Eq. (7) the maximum likelihood estimates of θ is expressed by

$$\hat{\theta }=r\;\;Q_{\hat{\lambda }}^{-1}\left(x;r,\;C,\;R\right),$$ (9)

where; $Q_{\hat{\lambda }}\left(x;r,\;C,\;R\right)=-\left[\sum_{i=1}^{r}\left(1+R_{i}\right)ln\left(1-e^{-\hat{\lambda }x_{i}^{-2}}\right)\;+R_{T}\;\;ln\left(1-e^{-\hat{\lambda }C^{-2}}\right)\;\right]$.

Consequently, by substituting θ into Eq. (8), the system equation reduced to one nonlinear equation as follows:


$$\frac{r}{\hat{\lambda }}-\sum_{i=1}^{r}x_{i}^{-2}+\sum_{i=1}^{r}\left[r\;Q_{\hat{\lambda }}^{-1}\left(x;r,\;C,\;R\right)\left(1+R_{i}\right)-1\right]\;x_{i}^{-2}\;\;e^{-\hat{\lambda }x_{i}^{-2}}{\left(1-e^{-\hat{\lambda }x_{i}^{-2}}\right)}^{-1}$$

$$+\left[r\;Q_{\hat{\lambda }}^{-1}\left(x;r,\;C,\;R\right)\right]R_{T}\;\;C^{-2}\;\;e^{-\hat{\lambda }C^{-2}}{\left(1-e^{-\hat{\lambda }C^{-2}}\right)}^{-1}=0.$$ (10)

It is seen that there is no analytical solution for Eq. (10). As a result, it is impossible to obtain the MLEs $\hat{\lambda }$ in their explicit form. Some numerical methods, such the Newton-Raphson method, can be used to get the necessary estimations to get around this problem; for more information about the ML approach. After obtaining the MLE $\hat{\lambda }$, the MLE $\hat{\theta }$ can be calculated using Eq. (9) by replacing λ with their MLE. Following the derivation of $\hat{\lambda }$ and $\hat{\theta }$, the MLEs of *R*(*x*) and *H*(*x*) may be obtained from Eqs. (3) and (4), respectively. This is accomplished by utilizing the invariance property of the MLEs $\hat{\lambda }$ and $\hat{\theta }$, as described in the following manner:


$$\hat{R}\left(x;\hat{\theta },\hat{\lambda }\right)={\left[1-\exp\left(-\hat{\lambda }x^{-2}\right)\right]}^{\hat{\theta }},$$

and


$$\hat{H}\left(x;\hat{\theta },\hat{\lambda }\right)=2\;\hat{\theta }\;\hat{\lambda }\;x^{-3}\;\exp\left(-\hat{\lambda }x^{-2}\right)\;{\left[1-\exp\left(-\hat{\lambda }x^{-2}\right)\right]}^{-1}.$$

To derive asymptotic confidence intervals (ACIs) for the unknown parameters $\xi \left(\varphi \right)={\left(\theta ,\;\lambda \right)}^{T}$, we rely on the asymptotic properties of the MLEs. According to large sample theory, the MLEs $\hat{\xi }\left(\varphi \right)={\left(\hat{\theta },\;\hat{\lambda }\right)}^{T}$ follow a normal distribution with a mean of φ and a variance-covariance matrix ${\text{I}}^{-1}\left(\varphi \right)$. We utilize the asymptotic variance-covariance matrix ${\text{I}}^{-1}\left(\hat{\xi }\left(\varphi \right)\right)$ to estimate ${\text{I}}^{-1}\left(\xi \left(\varphi \right)\right)$, which is obtained by inverting the observed Fisher information matrix; for further details on Fisher information and related information measures, see Husseiny et al. [43], Barakat et al. [44], and Alawady et al. [45]. In this scenario, the asymptotic variance-covariance matrix is expressed as:


$${\text{I}}^{-1}\left(\hat{\xi }\left(\varphi \right)\right)={\left(\begin{array}{cc} -\frac{\partial^{2}lnL\;}{\partial \theta^{2}} & -\frac{\partial lnL\;}{\partial \theta \;\partial \lambda }\\ -\frac{\partial lnL\;}{\partial \lambda \;\partial \theta } & -\frac{\partial^{2}lnL\;}{\partial \lambda^{2}} \end{array}\right)}^{-1}=\left(\begin{array}{cc} {\hat{\sigma }}_{11}^{2} & {\hat{\sigma }}_{12}\\ {\hat{\sigma }}_{21} & {\hat{\sigma }}_{22}^{2} \end{array}\right)$$

where the hat implies that the derivatives are evaluated at $\hat{\xi }\left(\varphi \right)=\left(\hat{\theta },\;\hat{\lambda }\right)$ and


$$\frac{\partial^{2}lnL\;}{\partial \theta^{2}}\;\;=\;\;-\frac{r}{\theta^{2}},$$

$$\frac{\partial lnL\;}{\partial \lambda \partial \theta }\;\;=\;\;\sum_{i=1}^{r}\left(1+R_{i}\right)\;x_{i}^{-2}\;\;e^{-\lambda x_{i}^{-2}}{\left(1-e^{-\lambda x_{i}^{-2}}\right)}^{-1}+R_{T}\;\;C^{-2}\;e^{-\lambda C^{-2}}{\left(1-e^{-\lambda C^{-2}}\right)}^{-1},$$

and

$$\frac{\partial^{2}lnL\;}{\partial \lambda^{2}}\;\;=\;\;-\frac{r}{\lambda^{2}}-\theta \;\;R_{T}\;\;C^{-4}\;\;e^{-\lambda C^{-2}}{\left(1-e^{-\lambda C^{-2}}\right)}^{-1}\left\{1+e^{-\lambda C^{-2}}{\left(1-e^{-\lambda C^{-2}}\right)}^{-1}\right\}$$

$$\;\;-\;\;\sum_{i=1}^{r}\left[\theta \left(1+R_{i}\right)-1\right]x_{i}^{-4}\;\;e^{-\lambda y_{i}^{-2}}{\left(1-e^{-\lambda y_{i}^{-2}}\right)}^{-1}\left\{1+e^{-\lambda y_{i}^{-2}}{\left(1-e^{-\lambda y_{i}^{-2}}\right)}^{-1}\right\}.$$

Utilizing the asymptotic normality of the MLEs, the 100(1−γ)% ACIs of ξ(φ)=(θ, λ) can be constructed, respectively, as

$\hat{\theta }\pm Z_{\gamma /2}\;{\hat{\sigma }}_{11}$ and $\hat{\lambda }\pm Z_{\gamma /2}\;{\hat{\sigma }}_{22}$,

where $Z_{\gamma /2}$ is the upper (γ/2) the percentile points of the standard normal distribution.

It is necessary to estimate the variances of the estimators of *R*(*x*) and *H*(*x*) to construct the 100(1−γ)% ACIs of both of these variables. A notable strategy used to approximate the variance of unknown parametric functions is the delta method, one of the most used and significant strategies. The approximated variances of the estimators of *R*(*x*) and *H*(*x*) can be derived in the following manner, respectively, by applying the delta method:

$$\hat{\upsilon }(\hat{R})\approx \left[\psi_{R}\;{𝐈}^{-1}(\hat{\theta },\hat{\lambda })\;\psi_{R}^{T}\right]\;\;\;and\;\;\;\hat{\upsilon }(\hat{H})\approx \left[\psi_{H}\;{𝐈}^{-1}(\hat{\theta },\hat{\lambda })\;\psi_{H}^{T}\right].$$ (11)

First, we must obtain $\psi_{R}=\left(\frac{\partial R}{\partial \theta },\frac{\partial R}{\partial \lambda }\right){|}_{\left(\hat{\theta },\hat{\lambda }\right)}$ and $\psi_{H}=\left(\frac{\partial R}{\partial \theta },\frac{\partial H}{\partial \lambda }\right){|}_{\left(\hat{\theta },\hat{\lambda }\right)}$ as follows:


$$\frac{\partial R}{\partial \theta }\;\;=\;\;\;{\left[1-e^{-\lambda x^{-2}}\right]}^{\theta }\;\;ln\left[1-e^{-\lambda x^{-2}}\right]\;,$$

$$\frac{\partial R}{\partial \lambda }\;\;=\;\;\;\theta \;\;x^{-2}\;\;e^{-\lambda x^{-2}}\;\;{\left[1-e^{-\lambda x^{-2}}\right]}^{\theta -1}\;\;,$$

$$\frac{\partial H}{\partial \theta }\;\;=\;\;\;2\;\lambda \;\;x^{-3}\;\;e^{-\lambda x^{-2}}\;\;{\left[1-e^{-\lambda x^{-2}}\right]}^{-1}\;\;,$$

and

$$\frac{\partial H}{\partial \lambda }\;\;=\;\;\;2\;x^{-3}\left\{e^{-\lambda x^{-2}}\left[\left(1-\lambda x^{-2}\right)\left(1-e^{-\lambda x^{-2}}\right)-\lambda x^{-2}\;\;e^{-\lambda x^{-2}}\right]\right\}{\left[1-e^{-\lambda x^{-2}}\right]}^{-2}\;.$$

Therefore, by employing the confidence level 100(1−γ)%, the two-sided ACIs for *R*(*x*) and *H*(*x*) are constructed in the following manner:

$\hat{R}(x)\pm Z_{\gamma /2}\;\sqrt{\hat{\upsilon }(\hat{R})}$     and     $\hat{H}(x)\pm Z_{\gamma /2}\;\sqrt{\hat{\upsilon }(\hat{H})}$.

## 3 Maximum product of spacing estimation

The MPSEs are achieved by selecting parameter values that optimize the product of the distances between the CDF values at adjacent ordered points. The MPSEs demonstrate superior efficacy for small sample sizes compared to the MLEs, rendering the MPS approach increasingly attractive in reliability and life testing studies. Numerous authors have regarded the MPS technique for estimating the unknown parameters of lifespan models; refer to Basu et al. [46]. This section proposes the MPS approach to derive point estimates and ACIs of the IER distribution based on PHT-ICS samples. The observed data may conform to one of two potential scenarios about the censoring scheme:

case I: $\left\{X_{1:m:n},\;X_{2:m:n},\;\ldots ,\;X_{m:m:n}\right\}$, if *X*_*m*:*m*:*n*_<*T*,

case II: $\left\{X_{1:m:n},\;X_{2:m:n},\;\ldots ,\;X_{d:m:n}\right\}$, if $X_{d:m:n}<T<X_{d+1:m:n}$.

However, we can write the MPSE function of the PHT-ICS as follows:

$$S\left[\xi \left(\varphi \right)|x\right]=B\;\prod_{i=1}^{r+1}\{F\left[x_{i};\xi \left(\varphi \right)\right]\;-\;F\left(x_{i-1};\xi \left(\varphi \right)\right)\}[\overset{―}{F}\left(x_{i};\xi \left(\varphi \right)\right){]}^{R_{i}}\;[\overset{―}{F}\left(C;\xi \left(\varphi \right)\right){]}^{R_{T}},$$ (12)

where *B* is the normalizing constant, and by convention $F\left(x_{0};\xi \left(\varphi \right)\right)=0,\;\;F\left(x_{r+1};\xi \left(\varphi \right)\right)=1,$ *r* = *m*, $C=x_{\left(m\right)}$, *R*_*T*_ = 0 in case I while case II, *r* = *d*, *C* = *T*, $R_{T}=\left(n-d-\sum_{i=1}^{d}R_{i}\right)$ and $x_{i}=x_{\left(i\right)}$. Then, from Eqs. (2) and (12), The MSPE, without the constant term, can be expressed as

$$S\left[\xi \left(\varphi \right)|x\right]\propto \;\prod_{i=1}^{r+1}\left[{\left(1-e^{-\lambda \;x_{i-1}^{-2}}\right)}^{\theta }-{\left(1-e^{-\lambda \;x_{i}^{-2}}\right)}^{\theta }\right]{\left(1-e^{-\lambda \;x_{i}^{-2}}\right)}^{\theta \;R_{i}}\;{\left(1-e^{-\lambda \;C^{-2}}\right)}^{\theta \;R_{T}}$$ (13)

Let s(ξ(φ)|x)=lnS(ξ(φ)|x) denoted by the natural logarithm of Eq. (13) as


$$s\left[\xi \left(\varphi \right)|x\right]=\sum_{i=1}^{r+1}\ln\left[{\left(1-e^{-\lambda \;x_{i-1}^{-2}}\right)}^{\theta }-{\left(1-e^{-\lambda \;x_{i}^{-2}}\right)}^{\theta }\right]+\theta \sum_{i=1}^{r+1}R_{i}\;\ln\left(1-e^{-\lambda \;x_{i}^{-2}}\right)$$

$$+\theta R_{T}\;\ln\left(1-e^{-\lambda \;C^{-2}}\right)$$ (14)

The MPSEs, represented by $\hat{\hat{\xi }}\left(\varphi \right)=\left(\hat{\hat{\theta }},\;\hat{\hat{\lambda }}\right)$, can be obtained by maximizing the objective function of Eq. (14) concerning ξ(φ)=(θ,λ). Simultaneously solving the following three nonlinear equations allows us to get the requisite estimators


$$\frac{\partial s\left[\xi \left(\varphi \right)|x\right]}{\partial \theta }=\sum_{i=1}^{r+1}{ℋ}_{i}^{-1}\left\{{𝔻}_{i-1}-{𝔻}_{i}\right\}+\sum_{i=1}^{r+1}R_{i}\ln\left(1-e^{-\lambda \;x_{i}^{-2}}\right)$$

$$+R_{T}\ln\left(1-e^{-\lambda \;C^{-2}}\right),$$ (15)

$$\frac{\partial s\left[\xi \left(\varphi \right)|x\right]}{\partial \lambda }=\theta \left\{\sum_{i=1}^{r+1}{ℋ}_{i}^{-1}\left[\phi_{i-1}-\phi_{i}\right]+\sum_{i=1}^{r+1}R_{i}\;\;\rho_{i}+R_{T}\;\;\rho^{*}\right\},$$ (16)

where, ${ℋ}_{i}=\ln\left\{(1-e^{-\lambda \;x_{i-1}^{-2}}{)}^{\theta }-(1-e^{-\lambda \;x_{i}^{-2}}{)}^{\theta }\right\}$, ${𝔻}_{i}=(1-e^{-\lambda \;x_{i}^{-2}}{)}^{\theta }\ln\left(1-e^{-\lambda \;x_{i}^{-2}}\right),$ $\phi_{i}=x_{i}^{-2}\;e^{-\lambda x_{i}^{-2}}(1-e^{-\lambda x_{i}^{-2}}{)}^{\theta -1}$, $\rho^{*}=C^{-2}\;e^{-\lambda C^{-2}}(1-e^{-\lambda C^{-2}}{)}^{-1}$ and $\rho_{i}=x_{i}^{-2}\;e^{-\lambda x_{i}^{-2}}(1-e^{-\lambda x_{i}^{-2}}{)}^{-1}$.

Similar to the MLEs, numerical methods should be employed to resolve Eqs. (15) and (16) in order to ascertain the MPSEs of ξ(φ)=(θ,λ). Cheng and Traylor [47] assert that the MPSEs exhibit the same invariance property as the MLEs. Consequently, we can get the MPSEs of *R*(*x*) and *H*(*x*) utilizing this condition as follows


$$\hat{\hat{R}}\left(x;\hat{\hat{\xi }}\left(\varphi \right)\right)={\left[1-\exp\left(-\hat{\hat{\lambda }}x^{-2}\right)\right]}^{\hat{\hat{\theta }}},$$

and


$$\hat{\hat{H}}\left(x;\hat{\hat{\xi }}\left(\varphi \right)\right)=2\;\hat{\hat{\theta }}\;\hat{\hat{\lambda }}\;x^{-3}\;\exp\left(-\hat{\hat{\lambda }}x^{-2}\right)\;{\left[1-\exp\left(-\hat{\hat{\lambda }}x^{-2}\right)\right]}^{-1}.$$

It may be noted that the MPSEs are represented as $\hat{\hat{\xi }}\left(\varphi \right)=\left(\hat{\hat{\theta }},\;\hat{\hat{\lambda }}\right)$ cannot be obtained in closed phrases. Consequently, any numerical method may be utilized to resolve Eqs. (15) and (16). We may derive the ACIs for the unknown parameters by utilizing the asymptotic qualities of MPSEs, analogous to our approach with MLEs. The asymptotic distribution of the MPSEs $\hat{\hat{\xi }}\left(\varphi \right)={\left(\hat{\hat{\theta }},\;\hat{\hat{\lambda }}\right)}^{T}$ follows a normal distribution with a mean of ξ(φ) and an asymptotic variance-covariance matrix ${\text{I}}^{-1}\left(\xi \left(\varphi \right)\right)$. We examine ${\text{I}}^{-1}\left(\hat{\hat{\xi }}\left(\varphi \right)\right)$ to approximate ${\text{I}}^{-1}\left(\xi \left(\varphi \right)\right)$, with the components of $\text{I}\left(\hat{\hat{\xi }}\left(\varphi \right)\right)$ detailed as follows

$$\frac{\partial^{2}s\left[\xi \left(\varphi \right)|x\right]}{\partial \theta^{2}}=\sum_{i=1}^{r+1}{ℋ}_{i}^{-1}\left({𝔻}_{i-1}^{\prime }-{𝔻}_{i}^{\prime }\right)-\sum_{i=1}^{r+1}{ℋ}_{i}^{-2}{\left({𝔻}_{i-1}-{𝔻}_{i}\right)}^{2},$$ (17)

$$\frac{\partial^{2}s\left[\xi \left(\varphi \right)|x\right]}{\partial \lambda^{2}}=\theta \sum_{i=1}^{r+1}x_{i-1}^{-2}\left\{\frac{\kappa_{i-1}}{{ℋ}_{i}}-\frac{\theta \;x_{i-1}^{2}\;\phi_{i-1}[\phi_{i-1}-\phi_{i}]}{{ℋ}_{i}^{2}}\right\}$$

$$-\theta \sum_{i=1}^{r+1}x_{i}^{-2}\left\{\frac{\kappa_{i}}{{ℋ}_{i}}-\frac{\theta \;x_{i}^{2}\;\phi_{i}\left[\phi_{i-1}-\phi_{i}\right]}{{ℋ}_{i}^{2}}\right\}$$

$$-\theta \sum_{i=1}^{r+1}R_{i}\;\;x_{i}^{-2}\left[\frac{x_{i}^{-2}e^{-2\lambda x_{i}^{-2}}}{{\left(1-e^{-\lambda x_{i}^{-2}}\right)}^{2}}+\frac{x_{i}^{-2}\;\;e^{-\lambda x_{i}^{-2}}}{\left(1-e^{-\lambda x_{i}^{-2}}\right)}\right]$$

$$-\theta \;R_{T}\;\;C^{-2}\left[\frac{C^{-2}\;\;e^{-2\lambda C^{-2}}}{{\left(1-e^{-\lambda C^{-2}}\right)}^{2}}+\frac{C^{-2}\;\;e^{-\lambda C^{-2}}}{\left(1-e^{-\lambda C^{-2}}\right)}\right],$$ (18)

$$\frac{\partial^{2}s\left[\xi \left(\varphi \right)|x\right]}{\partial \theta \partial \lambda }=\sum_{i=1}^{r+1}\left\{\frac{\phi_{i-1}\left[\theta \ln\left(1-e^{-\lambda x_{i-1}^{-2}}\right)+1\right]}{{ℋ}_{i}}-\frac{\theta \phi_{i-1}\left[{𝔻}_{i-1}-{𝔻}_{i}\right]}{{ℋ}_{i}^{2}}\right\}$$

$$-\sum_{i=1}^{r+1}\left\{\frac{\phi_{i}\left[\theta \ln\left(1-e^{-\lambda x_{i}^{-2}}\right)+1\right]}{{ℋ}_{i}}-\frac{\theta \phi_{i}\left[{𝔻}_{i-1}-{𝔻}_{i}\right]}{{ℋ}_{i}^{2}}\right\}$$

$$+R_{T}\rho^{*}+\sum_{i=1}^{r+1}R_{i}\rho_{i},$$ (19)

where, ${𝔻}_{i}^{\prime }=(1-e^{-\lambda \;x_{i}^{-2}}{)}^{\theta }\ln(1-e^{-\lambda \;x_{i}^{-2}}{)}^{2}$, and $\kappa_{i}=\phi_{i}[(\theta -1)\;e^{-\lambda x_{i}^{-2}}(1-e^{-\lambda x_{i}^{-2}}{)}^{-1}-1].$

After obtaining the estimated variances of $\hat{\hat{\xi }}\left(\varphi \right)=\left(\hat{\hat{\theta }},\;\hat{\hat{\lambda }}\right)$, denoted by ${\hat{\hat{\sigma }}}_{ij},i,j=1,2,3$ and i≠j which are the main diagonal elements of ${\text{I}}^{-1}\left(\hat{\hat{\xi }}\left(\varphi \right)\right)$, the 100(1−γ) % ACIs of ξ(φ)=(θ,λ) can be obtained, respectively, as follows

$\hat{\hat{\theta }}\pm Z_{\gamma /2}\;{\hat{\hat{\sigma }}}_{11}$, and $\hat{\hat{\lambda }}\pm Z_{\gamma /2}\;{\hat{\hat{\sigma }}}_{22}$

where $Z_{\gamma /2}$ is the upper (γ/2)th percentile point of the standard normal distribution.

By approximating the estimated variances of the RF and HRF using the delta method, we can obtain the 100(1−γ) % ACIs of *R*(*x*) and *H*(*x*), respectively, as follows

$\hat{\hat{R}}\left(x\right)\pm Z_{\gamma /2}\;\sqrt{\hat{\hat{\upsilon }}(\hat{\hat{R}})}$     and     $\hat{\hat{H}}(x)\pm Z_{\gamma /2}\;\sqrt{\hat{\hat{\upsilon }}(\hat{\hat{H}})}$,

where $\hat{\hat{\upsilon }}(\hat{\hat{R}})$ and $\hat{\hat{\upsilon }}(\hat{\hat{H}})$ are evaluated at the MPSEs of θ and λ as defined in Eq. (11).

## 4 Bayesian estimation

This section will derive the Bayes estimate using the square error loss function, assuming a gamma prior for the unknown parameters of the IER distribution. The estimation will be based on PHT-ICS. Bayesian estimation is performed assuming that the random variables θ and λ are independently distributed with a gamma prior distribution. Gamma priors are chosen for θ and λ due to their support on (0,∞), conjugacy with exponential family likelihoods, and the ability to encode prior mean/variance flexibly. They are a standard choice in Bayesian reliability (e.g., Nagy et al. [48]). The gamma prior distribution is characterised by known shape and scale parameters a, c, b, and d, and has a probability density function as


$$\pi \left(\theta \right)\propto \theta^{a-1}exp\left(-\theta b\right)\;;\;\;a,b>0,$$

and


$$\pi \left(\lambda \right)\propto \lambda^{c-1}exp\left(-\lambda d\right)\;;\;\;c,d>0.$$

The joint prior density of unknown parameters ξ(φ)=(θ,λ) is thus stated as

$$\pi \left(\xi \left(\varphi \right)\right)\propto \theta^{a-1}\lambda^{c-1}exp\left[-\left(\theta b+\lambda d\right)\right]\;;\;\;a,b,c,d>0.$$ (20)

Combining Eqs. (6) and (20) to obtain the posterior density of ξ(φ) take the following form:


$$\pi^{*}\left(\xi \left(\varphi )\right|\underset{―}{x}\right)={𝔼}^{-1}\theta^{r+a-1}\lambda^{r+c-1}{\left(1-e^{-\lambda C^{-2}}\right)}^{\theta R_{T}}exp\left\{-\left[\theta b+\lambda \left(d+\sum_{i=1}^{r}x_{i}^{-2}\right)\right]\right\}$$

$$\times \prod_{i=1}^{r}\left[{\left(1-e^{-\lambda x_{i}^{-2}}\right)}^{\theta \left(1+R_{i}\right)-1}\right],$$ (21)

where 𝔼 is the normalized constant and is provided by


$$𝔼=\int_{0}^{\infty }\int_{0}^{\infty }\theta^{a-1}\lambda^{c-1}{\left(1-e^{-\lambda C^{-2}}\right)}^{\theta R_{T}}exp\left\{-\left[\theta b+\lambda \left(d+\sum_{i=1}^{r}x_{i}^{-2}\right)\right]\right\}\;$$

$$\times \prod_{i=1}^{r}\left[{\left(1-e^{-\lambda x_{i}^{-2}}\right)}^{\theta \left(1+R_{i}\right)-1}\right]\;\;\;d\theta \;d\lambda$$

Bayesian analysis relies on the loss function to assess overestimation and underestimation. Symmetric and asymmetric loss functions are frequent. The symmetric loss function weights overestimation and underestimation equally, whereas the asymmetric loss function weights them differently. We examine the popular symmetric SE loss function and the asymmetric LINEX loss function in this paper. The posterior mean is the Bayes estimator for SE loss functions. Under SE loss function, the posterior mean is the best estimate. However, the LINEX loss function lets you weight overestimation and underestimation differently, representing their relative relevance.

Varian [49] created the asymmetric LINEX loss function, which mixes exponential and linear growth on either side of zero. It seeks to capture overestimation and underestimation asymmetry. Based on the premise that the minimal loss occurs at $\tilde{\tilde{\xi }}\left(\varphi \right)=\xi (\varphi )$, the LINEX loss function as


$$L\left[\Delta )\right]\;\;\propto \;\;e^{a\Delta }-a\Delta -1\;,\;\;a\neq 0\;,$$

where, $\Delta =\left[\tilde{\tilde{\xi }}\left(\varphi \right)-\xi (\varphi ))\right]$, $\tilde{\tilde{\xi }}\left(\varphi \right)$ is an estimate of ξ(φ). The constant parameter (a) determines the shape of the loss function. If the parameter *a* is greater than 0 and the error $\left[\tilde{\tilde{\xi }}\left(\varphi \right)-\xi (\varphi )\right]$ is considered.The LINEX loss function has an almost exponential behaviour for positive mistakes and an almost linear behaviour for negative errors. In such scenarios, over-estimations pose a more significant issue than under-estimations. If *a*<0, underestimation is prioritised over overestimation. When the absolute value of *a* is small, the loss function is almost symmetric and behaves similarly to the squared error (SE) loss function. The Bayes estimates of ξ(φ) under the LINEX loss function are provided by:


$$\tilde{\tilde{\xi }}\left(\varphi \right)=-\frac{1}{a}\;ln\;E\left(e^{-a\xi (\varphi )}|\underset{―}{x}\right)\;,\;\;\;\;a\;\neq \;0\;,$$

provided that $E\left(e^{-a\xi (\varphi )}|\underset{―}{x}\right)$ exists, and is finite (see Zellner [50]).

Therefore, the Bayes estimators of the unknown parameters ξ(φ)=(θ, λ) under PHT-ICS based on SE and LINEX loss functions, denoted by ${\tilde{\xi }\left(\varphi \right)}_{(BESL)}$ and ${\tilde{\tilde{\xi }}\left(\varphi \right)}_{\left(BELL\right)}$ respectively, can be obtained as follow:

$${\tilde{\xi }\left(\varphi \right)}_{\left(BESL\right)}=E\left[\left(\xi \left(\varphi )\right|\underset{―}{x}\right)\right]=\int_{0}^{\infty }\int_{0}^{\infty }\xi \left(\varphi \right)\pi^{*}\left(\xi \left(\varphi )\right|\underset{―}{x}\right)d\theta \;\;d\lambda \;,$$ (22)

and

$${\tilde{\tilde{\xi }}\left(\varphi \right)}_{\left(BELL\right)}=-\frac{1}{a}ln\left[\int_{0}^{\infty }\int_{0}^{\infty }e^{-a\xi \left(\varphi \right)}\;\pi^{*}\left(\xi \left(\varphi \right)|\underset{―}{x}\right)\;\;\;\;d\theta \;\;d\lambda \;\right]\;.$$ (23)

where $\pi^{*}\left(\xi \left(\varphi \right)|\underset{―}{x}\right)$ is the joint posterior distribution given by Eq. (21). Analytically calculating Bayes estimators using Eqs. (22) and (23) are impossible owing to integration difficulty. We recommend using Markov Chain Monte Carlo (MCMC) to provide Bayes estimates of unknown parameters and highest posterior density (HPD) credible intervals. To use the MCMC technique, we must determine the entire conditional distributions of the parameters ξ(φ)=(θ,λ). Based on Eq. (21), we can determine the necessary full conditional distributions as follows:


$$\pi^{*}\left(\xi \left(\varphi \right)|x\right)\propto \theta^{a+r-1}\lambda^{c+r-1}{\left(1-e^{-\lambda C^{-2}}\right)}^{\theta R_{T}}exp\left\{-\left[\theta b+\lambda \left(d+\sum_{i=1}^{r}x_{i}^{-2}\right)\right]\right\}\;$$

$$\times \left[\prod_{i=1}^{r}{\left(1-e^{-\lambda x_{i}^{-2}}\right)}^{\theta \left(1+R_{i}\right)-1}\right];\;\;\;a,\;b,\;c\;,\;d,\;x,\;\theta ,\;\lambda \;>\;0\;,\;$$ (24)

The conditional posterior densities of ξ(φ)=(θ, λ) are as follows:

$$\pi_{1}^{*}\left(\theta |\lambda ,\;x\right)\propto \theta^{a+r-1}\;\;e^{-\left(\theta b\right)}{\left(1-e^{-\lambda C^{-2}}\right)}^{\theta R_{T}}\left[\prod_{i=1}^{r}{\left(1-e^{-\lambda x_{i}^{-2}}\right)}^{\theta \left(1+R_{i}\right)-1}\right],$$ (25)

and

$$\pi_{2}^{*}\left(\lambda |\theta ,\;x\right)\propto \lambda^{c+r-1}\;\;e^{-\lambda \left(d+\sum_{i=1}^{r}x_{i}^{-2}\right)}{\left(1-e^{-\lambda C^{-2}}\right)}^{\theta R_{T}}\left[\prod_{i=1}^{r}{\left(1-e^{-\lambda x_{i}^{-2}}\right)}^{\theta \left(1+R_{i}\right)-1}\right].$$ (26)

By obtaining these full conditional distributions, we can employ MCMC methods, such as Gibbs sampling or Metropolis-Hasting’s algorithm, to iteratively sample from these distributions and obtain posterior samples of the parameters. The Bayes estimates and HPD credible intervals for unknown parameters can be estimated using these samples. We know the full conditional distributions for each parameter, but their forms are unknown, making direct sampling difficult. We create samples from these distributions using the Metropolis-Hastings (M-H) technique to address this.

The M-H sample proposal distribution is the normal distribution, which we use to calculate Bayesian estimates and HPD credible intervals. Using the entire conditional distributions in Eqs. (25) and (26), we can sketch the M-H algorithm steps:

**Step 1.** Initialization: Start with the first chain j = 1.

**Step 2.** Set the initial values of $\left(\theta^{\left(0\right)},\;\lambda^{\left(0\right)}\right)$ to $\left(\hat{\theta },\;\hat{\lambda }\right)$.

**Step 3.** Simulate $\theta^{\left(j\right)}$ using Eq. (25) and the Metropolis-Hastings (M-H) algorithm. Use a normal proposal distribution with mean $\theta^{\left(j-1\right)}$ and variance $\left[\hat{Var}\left(\theta \right)\right]$.

**Step 4.** Simulate $\lambda^{\left(j\right)}$ using Eq. (26) and the M-H algorithm. Use a normal proposal distribution with mean $\lambda^{\left(j-1\right)}$ and variance $\left[\hat{Var}\left(\lambda \right)\right]$.

**Step 5.** Replace θ and λ in Eqs. (3) and (4) with their respective $\theta^{\left(j\right)}$ and $\lambda^{\left(j\right)}$ to compute *R*^(*j*)^(*x*) and *H*^(*j*)^(*x*) for *x*>0 .

**Step 6.** Increment *j*: Set *j* = *j* + 1.

**Step 7.** Repeat Steps 3 to 6 a total of *N* times to obtain $\theta^{\left(j\right)},\lambda^{\left(j\right)}$, *R*^(*j*)^(*x*) and *H*^(*j*)^(*x*) for j=1, …, N.

The process entails sequentially sampling from the proposal distribution, determining the acceptance or rejection of the suggested samples based on the acceptance probability, and subsequently updating the parameter values. By iterating this process sufficiently, we can acquire a collection of posterior samples. From these samples, we may derive Bayesian estimates and establish the HPD credible intervals for the unknown values. To guarantee convergence and mitigate the impact of starting values, it is standard procedure to exclude the initial B samples produced by the MCMC chain. In this instance, we have acquired samples of $\theta^{\left(j\right)},\lambda^{\left(j\right)}$, *R*^(*j*)^(*x*), and *H*^(*j*)^(*x*) for j=B+1,…,N, where *N* denotes the total number of iterations. By eliminating the early *B* samples, we acquire a resultant sample that relies on a suitably substantial *B*. The resulting sample can be regarded as an approximate posterior sample, suitable for calculating Bayes estimates and the HPD credible intervals.

Using this approximate posterior sample, we can estimate the Bayes estimates for the unknown parameters by, for example, taking the mean or median of the sample. Additionally, we can construct the HPD credible intervals by identifying the range of parameter values that contain a specified proportion of the sample, such as the central 95% or 99% of the values. By discarding the initial samples and utilizing the resulting approximate posterior sample, we can obtain reliable estimates and credible intervals for the parameters of interest.

We can calculate the SE loss function based on Bayes estimate of ${\tilde{\xi }\left(\varphi \right)}_{(BESL)}$ can be calculated as


$${\tilde{\xi }\left(\varphi \right)}_{(BESL)}=\frac{1}{N-B}\sum_{j=B+1}^{N}{\left[\xi \left(\varphi \right)\right]}^{\left(j\right)}\;.$$

Similarly, we can use the following formula to obtain the Bayes estimate of ${\tilde{\tilde{\xi }}\left(\varphi \right)}_{\left(BELL\right)}$ based on the LINEX loss function.


$${\tilde{\tilde{\xi }}\left(\varphi \right)}_{\left(BELL\right)}=\frac{-1}{a}\left\{\frac{1}{N-B}\sum_{j=B+1}^{N}e^{-a\;{\left[\xi \left(\varphi \right)\right]}^{\left(j\right)}}\right\}\;.$$

To calculate the HPD credible intervals of the parameters θ and λ, denoted as ξ(φ), we can follow these steps:

**Step 1.** Arrang the samples of ξ(φ), denoted as ${\left[\xi \left(\varphi \right)\right]}^{\left(j\right)}$, in ascending order: ${\left[\xi \left(\varphi \right)\right]}^{\left(B+1\right)}<{\left[\xi \left(\varphi \right)\right]}^{\left(B+2\right)}<\ldots <{\left[\xi \left(\varphi \right)\right]}^{\left(N\right)}$.

**Step 2.** Define the index j^*^ as follows: $j^{*}=B+1,\;B+2,\;\ldots ,\;N$, such that:


$${\left[\xi \left(\varphi \right)\right]}^{\left(j^{*}+\left(1-\gamma \right)\left(N-B\right)\right)}-{\left[\xi \left(\varphi \right)\right]}^{\left(j^{*}\right)}=\underset{1\leq j\leq \;\gamma \left(N-B\right)}{min}\left\{{\left[\xi \left(\varphi \right)\right]}^{\left(j+\left[\left(1-\gamma \right)\left(N-B\right)\right]\right)}\right\}\;.$$

**Step 3.** The 100(1−γ)% HPD credible of ϖ is then given by:


$$\left\{{\left[\xi \left(\varphi \right)\right]}^{\left(j^{*}\right)},\;{\left[\xi \left(\varphi \right)\right]}^{\left(j^{*}+\left[\left(1-\gamma \right)\left(N-B\right)\right]\right)}\right\},$$

where [υ] denotes the highest number less than or equal to υ.

## 5 Monte Carlo simulations

Extensive Monte Carlo simulations are conducted to assess the performance of the suggested point and interval estimators for the life parameters θ, λ, *R*(*x*), and *H*(*x*). To achieve this purpose, the true parameter values of (θ, λ) were set at (0.4,0.8), and the progressive hybrid Type-I censoring was reproduced 1000 times. At mission time *t* = 0.5, the survival time estimations of *R*(*x*) and *H*(*x*) are assessed, with their actual values being 0.9834913 and 0.2175712, respectively. Each unknown parameter is estimated using several values of (*T*,*n*,*m*), specifically: (T=1.5,2.5), (n=40,80), and *m* is defined as a failure percentage for each *n*, so that $\frac{r}{n}\;\times \;100\%=50\%$ and 80%. Additionally, for each pair (*n*,*m*), various censoring strategies are examined, specifically


$$\text{Scheme-1}:\;R_{1}=n-m,\;R_{i}=0\;\text{for}\;i\neq 1;\;\text{Scheme-2}:R_{\frac{r}{2}}=n-m,\;R_{i}=0\;\text{for}\;i\neq \frac{m}{2};\;\text{Scheme-3}:R_{r}=n-m,\;R_{i}=0\;\text{for}\;i\neq m.$$

To run the experiment according to PHT-ICS from the proposed IER distribution, do the following procedure:

Step 1. Set the parameter values of θ and λ.
Step 2. Generate an ordinary progressive Type-II censored sample as:
  - Generate τ independent observations of size *m* as $\tau_{1},\tau_{2},...,\tau_{m}$.
  - For specific values of *n*, *m* and $R_{i},\;i=1,2,...,m$, set $\upsilon_{i}=\tau_{i}^{{\left(i+\sum_{j=r-i+1}^{r}R_{j}\right)}^{-1}},\;i=1,2,...,m$.
  - Set $U_{i}=1-\upsilon_{m}\upsilon_{m-1}\cdots \upsilon_{m-i+1}$ for i=1,2,...,m. Hence, $U_{i},\;i=1,2,...,m,$ is a progressive Type-II censored sample of size *m* from *U*(0,1) distribution.
  - Set $X_{i}=F^{-1}(u_{i};\theta ,\lambda ),\;i=1,2,...,m,$ is generated progressive Type-II censored sample from IER(θ,λ).
Step 3. Upon PHT-ICS, the sample data will consists of one of the following forms:
  - If *X*_*m*_<*T*, the experiment stops at *X*_*m*_ with observed failures $\left(X_{1},X_{2},\ldots ,X_{m}\right)$ and censoring $\left(R_{1},R_{2},\ldots ,R_{m}\right)$, that is Case-I.
  - If *T*<*X*_*m*_, the experiment stops at *T* with observed failures $\left(X_{1},X_{2},\ldots ,X_{D}\right)$ and censoring $\left(R_{1},R_{2},\ldots ,R_{D}\right)$, that is Case-II.

Upon the collection of 1,000 PHT-ICS, the maximum likelihood estimates (MLEs) and the corresponding 95% asymptotic confidence intervals (ACI) for θ, λ, *R*(*x*), and *H*(*x*) are computed using the R 4.2.2 programming language, following the installation of the ’maFxLik’ package developed by Henningsen and Toomet [51].To evaluate the impact of the proposed gamma priors on the Bayesian analysis, based on the prior mean and prior variance criteria established by Kundu [52], two distinct sets of informative hyperparameters (a, b, c, d) are employed, designated as Prior-1: (2, 5, 4, 5) and Prior-2: (4, 10, 8, 10). The values of *a*, *b*, *c*, and *d* are selected to ensure that the prior average equals the expected value of the unknown target parameter. In this instance, we conducted all Bayesian assessments utilizing informative priors, as the absence of prior information regarding the unknown parameter(s) renders the maximum likelihood approach more favorable than the Bayesian approach, which is more computationally intensive; for further details, refer to Dey and Elshahhat [53]. Using the R 4.2.2 programming language and the ’coda’ package recommended by Plummer et al. [54], we produce 12,000 MCMC samples, with the initial 2,000 iterations designated as burn-in. Consequently, the remaining 10,000 MCMC samples are employed to compute the Bayes point estimates for the SE and LINEX loss functions (for (q=−2,+2)), along with the 95% HPD credible interval estimates. The average point estimates (APEs) of θ are numerically represented as


$$\overset{―}{\overset{ˇ}{\theta }}=\frac{1}{1000}\sum_{i=1}^{1000}{\overset{ˇ}{\theta }}^{\left(i\right)},$$

where ${\overset{ˇ}{\theta }}^{\left(i\right)}$ is the estimate of θ at *i*th sample.

Comparison between point estimates of θ is conducted based on their root mean squared-errors (RMSEs) and mean relative absolute biases (MRABs) accordingly


$$\text{RMSE}(\overset{ˇ}{\theta })=\sqrt{\frac{1}{1000}\sum_{i=1}^{1000}{\left({\overset{ˇ}{\theta }}^{\left(i\right)}-\theta \right)}^{2}},$$

and


$$\text{MRAB}(\overset{ˇ}{\theta })=\frac{1}{1000}\sum_{i=1}^{1000}\frac{1}{\theta }\left|{\overset{ˇ}{\theta }}^{\left(i\right)}-\theta \right|.$$

On the other hand, the comparison between interval estimates of θ is made based on their average confidence lengths (ACLs) and coverage percentages (CPs) as


$${\text{ACL}}_{(1-\gamma )\%}(\theta )=\frac{1}{1000}\sum_{i=1}^{1000}\left({𝒰}_{{\overset{ˇ}{\theta }}^{\left(i\right)}}-{ℒ}_{{\overset{ˇ}{\theta }}^{\left(i\right)}}\right),$$

and


$${\text{CP}}_{(1-\gamma )\%}\left(\theta_{k}\right)=\frac{1}{1000}\sum_{i=1}^{1000}1_{\left({ℒ}_{{\overset{ˇ}{\theta }}^{\left(i\right)}};{𝒰}_{{\overset{ˇ}{\theta }}^{\left(i\right)}}\right)}\left(\theta_{k}\right),$$

respectively, where 1(·) is the indicator function, (ℒ(·),𝒰(·)) denote the (lower,upper) bounds of (1−γ)% asymptotic (or Bayes) interval estimate of θ. Clearly, in a similar fashion, the APEs, RMSEs, MRABs, ACLs, and CPs of λ, *R*(*x*), and *H*(*x*) can be easily obtained. All simulation results of θ, λ, *R*(*x*) and *H*(*x*) are reported in Tables 1–6.

**Table 1:** The APEs, RMSEs and MRABs of the MLE, MPS and BEs for θ based on the PHT-ICS under various censoring schemes.

*T*	(*n,m*) Sch.		MLE			MPS			Baysian								
									SE			LINEX					
									Prior-1			Prior-1					
									Prior-2			Prior-2					
q→												–2			+2		
			**APE**	**RMSE**	**MRAB**	**APE**	**RMSE**	**MRAB**	**APE**	**RMSE**	**MRAB**	**APE**	**RMSE**	**MRAB**	**APE**	**RMSE**	**MRAB**
1.5	(40,20)	1	0.4746	0.2436	0.3885	0.4334	0.1987	0.3371	0.4212	0.1199	0.2230	0.4409	0.1350	0.2453	0.4044	0.1099	0.2100
									0.4112	0.0915	0.1747	0.4247	0.0988	0.1856	0.3990	0.0866	0.1683
		2	0.5097	0.2861	0.4665	0.4600	0.2379	0.4004	0.4281	0.1185	0.2241	0.4556	0.1406	0.2619	0.4058	0.1049	0.2032
									0.4143	0.0877	0.1696	0.4317	0.0981	0.1876	0.3991	0.0814	0.1598
		3	0.4870	0.3013	0.4817	0.4545	0.2627	0.4278	0.4104	0.1199	0.2339	0.4394	0.1374	0.2628	0.3870	0.1109	0.2213
									0.4021	0.0897	0.1775	0.4208	0.0974	0.1899	0.3857	0.0862	0.1729
1.5	(40,32)	1	0.4531	0.1969	0.3336	0.4200	0.1719	0.3021	0.4131	0.1035	0.1995	0.4314	0.1146	0.2175	0.3974	0.0966	0.1891
									0.4064	0.0813	0.1598	0.4193	0.0870	0.1688	0.3947	0.0779	0.1551
		2	0.4612	0.2075	0.3478	0.4254	0.1775	0.3084	0.4144	0.1034	0.2050	0.4334	0.1152	0.2252	0.3981	0.0960	0.1925
									0.4064	0.0813	0.1637	0.4197	0.0874	0.1743	0.3944	0.0778	0.1577
		3	0.4482	0.2024	0.3601	0.4149	0.1776	0.3255	0.4057	0.1076	0.2098	0.4264	0.1189	0.2300	0.3882	0.1013	0.1999
									0.3999	0.0842	0.1657	0.4142	0.0895	0.1759	0.3870	0.0815	0.1615
1.5	(80,40)	1	0.4334	0.1238	0.2331	0.4110	0.1123	0.2177	0.4167	0.0902	0.1754	0.4273	0.0966	0.1862	0.4069	0.0853	0.1676
									0.4106	0.0746	0.1462	0.4190	0.0786	0.1531	0.4028	0.0716	0.1414
		2	0.4566	0.1657	0.2998	0.4258	0.1454	0.2698	0.4272	0.1044	0.2019	0.4434	0.1171	0.2236	0.4131	0.0952	0.1865
									0.4175	0.0815	0.1604	0.4292	0.0887	0.1734	0.4069	0.0762	0.1514
		3	0.4403	0.1616	0.2987	0.4154	0.1460	0.2771	0.4172	0.1063	0.2062	0.4347	0.1171	0.2240	0.4019	0.0992	0.1954
									0.4107	0.0851	0.1668	0.4236	0.0913	0.1770	0.3989	0.0811	0.1609
1.5	(80,64)	1	0.4284	0.1215	0.2301	0.4064	0.1117	0.2167	0.4141	0.0912	0.1780	0.4245	0.0972	0.1877	0.4046	0.0868	0.1711
									0.4092	0.0764	0.1506	0.4174	0.0802	0.1568	0.4014	0.0736	0.1465
		2	0.4304	0.1246	0.2344	0.4079	0.1138	0.2193	0.4152	0.0923	0.1799	0.4259	0.0989	0.1904	0.4054	0.0875	0.1724
									0.4099	0.0769	0.1518	0.4184	0.0811	0.1587	0.4020	0.0738	0.1472
		3	0.4252	0.1300	0.2431	0.4023	0.1198	0.2307	0.4091	0.0932	0.1812	0.4212	0.0996	0.1913	0.3981	0.0889	0.1751
									0.4041	0.0776	0.1528	0.4135	0.0813	0.1586	0.3954	0.0752	0.1495
2.5	(40,20)	1	0.4838	0.2317	0.3527	0.4307	0.1783	0.2836	0.4344	0.1186	0.2171	0.4506	0.1348	0.2419	0.4203	0.1066	0.1988
									0.4220	0.0910	0.1728	0.4335	0.0998	0.1878	0.4115	0.0842	0.1619
		2	0.4666	0.2023	0.3504	0.4266	0.1705	0.3104	0.4220	0.1117	0.2131	0.4420	0.1268	0.2362	0.4048	0.1017	0.1987
									0.4122	0.0863	0.1679	0.4262	0.0943	0.1811	0.3996	0.0810	0.1601
		3	0.4454	0.1843	0.3343	0.4180	0.1620	0.3028	0.4123	0.1115	0.2182	0.4310	0.1231	0.2362	0.3959	0.1041	0.2072
									0.4061	0.0883	0.1752	0.4200	0.0949	0.1853	0.3936	0.0844	0.1696
2.5	(40,32)	1	0.4278	0.1215	0.2358	0.4049	0.1109	0.2192	0.4100	0.0896	0.1786	0.4206	0.0951	0.1876	0.4002	0.0857	0.1722
									0.4059	0.0762	0.1522	0.4144	0.0798	0.1585	0.3979	0.0738	0.1481
		2	0.4494	0.1518	0.2781	0.4217	0.1340	0.2536	0.4229	0.1017	0.1968	0.4359	0.1107	0.2110	0.4112	0.0948	0.1864
									0.4157	0.0828	0.1621	0.4257	0.0886	0.1714	0.4064	0.0785	0.1553
		3	0.4386	0.1441	0.2677	0.4134	0.1295	0.2455	0.4147	0.0986	0.1914	0.4277	0.1063	0.2045	0.4030	0.0931	0.1822
									0.4093	0.0816	0.1602	0.4194	0.0864	0.1688	0.3999	0.0782	0.1545
2.5	(80,40)	1	0.4356	0.1095	0.1985	0.4083	0.0942	0.1770	0.4219	0.0844	0.1591	0.4297	0.0904	0.1687	0.4146	0.0794	0.1514
									0.4163	0.0715	0.1373	0.4228	0.0757	0.1441	0.4102	0.0680	0.1317
		2	0.4369	0.1283	0.2352	0.4123	0.1145	0.2170	0.4202	0.0941	0.1800	0.4313	0.1018	0.1922	0.4100	0.0882	0.1712
									0.4143	0.0775	0.1504	0.4232	0.0825	0.1587	0.4060	0.0736	0.1444
		3	0.4312	0.1215	0.2275	0.4118	0.1112	0.2125	0.4166	0.0922	0.1770	0.4273	0.0984	0.1870	0.4068	0.0874	0.1698
									0.4113	0.0776	0.1501	0.4199	0.0818	0.1570	0.4032	0.0745	0.1453
2.5	(80,64)	1	0.4158	0.0810	0.1562	0.4012	0.0764	0.1500	0.4089	0.0688	0.1345	0.4146	0.0713	0.1386	0.4035	0.0667	0.1313
									0.4068	0.0614	0.1208	0.4118	0.0634	0.1240	0.4020	0.0599	0.1184
		2	0.4188	0.0902	0.1712	0.4020	0.0841	0.1630	0.4104	0.0745	0.1439	0.4171	0.0779	0.1492	0.4041	0.0718	0.1399
									0.4078	0.0657	0.1280	0.4135	0.0682	0.1321	0.4023	0.0637	0.1248
		3	0.4164	0.0920	0.1785	0.4000	0.0867	0.1702	0.4086	0.0766	0.1504	0.4155	0.0798	0.1559	0.4021	0.0741	0.1461
									0.4060	0.0677	0.1340	0.4119	0.0700	0.1382	0.4004	0.0659	0.1308

**Table 2:** The APEs, RMSEs and MRABs of the MLE, MPS and BEs for λ based on the PHT-ICS under various censoring schemes.

*T*	(*n,m*) Sch.		MLE			MPS			Baysian								
									SE			LINEX					
									Prior-1			Prior-1					
									Prior-2			Prior-2					
q→												–2			+2		
			**APE**	**RMSE**	**MRAB**	**APE**	**RMSE**	**MRAB**	**APE**	**RMSE**	**MRAB**	**APE**	**RMSE**	**MRAB**	**APE**	**RMSE**	**MRAB**
1.5	(40,20)	1	0.9231	0.3821	0.3311	0.7860	0.3226	0.3024	0.8342	0.1811	0.1744	0.8938	0.2237	0.2107	0.7827	0.1589	0.1588
									0.8204	0.1283	0.1260	0.8610	0.1524	0.1482	0.7839	0.1169	0.1168
		2	0.9406	0.3918	0.3436	0.7974	0.3272	0.3046	0.8314	0.1676	0.1657	0.8929	0.2108	0.2031	0.7782	0.1470	0.1480
									0.8170	0.1189	0.1195	0.8581	0.1429	0.1415	0.7800	0.1088	0.1099
		3	0.9403	0.4657	0.3826	0.7932	0.3960	0.3470	0.8347	0.1844	0.1782	0.9065	0.2376	0.2265	0.7753	0.1597	0.1585
									0.8214	0.1304	0.1286	0.8675	0.1585	0.1564	0.7810	0.1181	0.1175
1.5	(40,32)	1	0.8960	0.3777	0.3172	0.7663	0.3291	0.3035	0.8201	0.1785	0.1731	0.8778	0.2175	0.2037	0.7700	0.1600	0.1621
									0.8102	0.1276	0.1267	0.8499	0.1490	0.1448	0.7746	0.1190	0.1206
		2	0.9232	0.3867	0.3282	0.7873	0.3277	0.2969	0.8342	0.1801	0.1752	0.8944	0.2237	0.2128	0.7823	0.1574	0.1572
									0.8211	0.1289	0.1278	0.8622	0.1538	0.1512	0.7842	0.1170	0.1176
		3	0.8932	0.3889	0.3305	0.7549	0.3379	0.3126	0.8179	0.1753	0.1720	0.8795	0.2158	0.2053	0.7653	0.1580	0.1598
									0.8095	0.1247	0.1243	0.8512	0.1468	0.1437	0.7723	0.1166	0.1179
1.5	(80,40)	1	0.8679	0.2447	0.2275	0.7864	0.2206	0.2165	0.8323	0.1676	0.1633	0.8670	0.1895	0.1800	0.8003	0.1532	0.1526
									0.8228	0.1322	0.1308	0.8499	0.1473	0.1426	0.7974	0.1228	0.1236
		2	0.8726	0.2475	0.2329	0.7847	0.2219	0.2200	0.8275	0.1582	0.1553	0.8643	0.1800	0.1736	0.7936	0.1451	0.1456
									0.8176	0.1224	0.1213	0.8456	0.1367	0.1341	0.7915	0.1145	0.1149
		3	0.8471	0.2564	0.2388	0.7482	0.2367	0.2355	0.8151	0.1600	0.1564	0.8586	0.1842	0.1763	0.7766	0.1483	0.1483
									0.8083	0.1227	0.1210	0.8404	0.1376	0.1332	0.7791	0.1164	0.1168
1.5	(80,64)	1	0.8521	0.2257	0.2106	0.7713	0.2075	0.2052	0.8221	0.1571	0.1524	0.8560	0.1766	0.1683	0.7909	0.1454	0.1443
									0.8152	0.1245	0.1225	0.8418	0.1377	0.1338	0.7903	0.1170	0.1170
		2	0.8539	0.2268	0.2116	0.7726	0.2080	0.2050	0.8228	0.1572	0.1522	0.8569	0.1768	0.1686	0.7915	0.1453	0.1439
									0.8156	0.1243	0.1220	0.8423	0.1377	0.1337	0.7906	0.1167	0.1163
		3	0.8561	0.2468	0.2247	0.7674	0.2243	0.2178	0.8235	0.1633	0.1573	0.8616	0.1864	0.1749	0.7890	0.1498	0.1482
									0.8161	0.1274	0.1245	0.8451	0.1425	0.1363	0.7892	0.1191	0.1189
2.5	(40,20)	1	0.9379	0.3587	0.3143	0.7766	0.2874	0.2744	0.8509	0.1880	0.1786	0.9069	0.2306	0.2162	0.8020	0.1620	0.1593
									0.8307	0.1343	0.1308	0.8700	0.1595	0.1545	0.7952	0.1201	0.1191
		2	0.9089	0.3406	0.3007	0.7762	0.2906	0.2768	0.8312	0.1752	0.1686	0.8884	0.2150	0.2022	0.7814	0.1547	0.1541
									0.8176	0.1253	0.1236	0.8573	0.1482	0.1440	0.7819	0.1149	0.1154
		3	0.8905	0.3689	0.3278	0.7526	0.3196	0.3070	0.8268	0.1899	0.1843	0.8909	0.2340	0.2196	0.7734	0.1691	0.1692
									0.8141	0.1351	0.1336	0.8573	0.1586	0.1536	0.7760	0.1254	0.1265
2.5	(40,32)	1	0.8834	0.3084	0.2748	0.7667	0.2700	0.2602	0.8302	0.1824	0.1748	0.8825	0.2189	0.2040	0.7845	0.1623	0.1607
									0.8185	0.1348	0.1318	0.8563	0.1568	0.1509	0.7844	0.1237	0.1235
		2	0.8996	0.3259	0.2919	0.7794	0.2824	0.2696	0.8363	0.1870	0.1800	0.8897	0.2248	0.2119	0.7896	0.1654	0.1630
									0.8213	0.1363	0.1338	0.8595	0.1587	0.1544	0.7869	0.1247	0.1240
		3	0.8953	0.3414	0.3011	0.7697	0.2959	0.2814	0.8340	0.1887	0.1818	0.8906	0.2291	0.2154	0.7853	0.1666	0.1656
									0.8205	0.1377	0.1361	0.8603	0.1610	0.1567	0.7848	0.1261	0.1268
2.5	(80,40)	1	0.8729	0.2280	0.2105	0.7802	0.1998	0.1968	0.8413	0.1666	0.1594	0.8726	0.1873	0.1761	0.8122	0.1519	0.1487
									0.8303	0.1344	0.1309	0.8556	0.1492	0.1435	0.8066	0.1242	0.1232
		2	0.8558	0.2186	0.2069	0.7758	0.2006	0.1992	0.8243	0.1553	0.1510	0.8570	0.1737	0.1663	0.7939	0.1441	0.1428
									0.8168	0.1241	0.1218	0.8428	0.1370	0.1329	0.7924	0.1168	0.1163
		3	0.8698	0.2606	0.2403	0.7764	0.2328	0.2312	0.8391	0.1796	0.1723	0.8797	0.2067	0.1934	0.8030	0.1624	0.1605
									0.8272	0.1403	0.1368	0.8582	0.1581	0.1511	0.7989	0.1295	0.1291
2.5	(80,64)	1	0.8423	0.1944	0.1818	0.7717	0.1808	0.1792	0.8222	0.1501	0.1436	0.8508	0.1661	0.1567	0.7956	0.1399	0.1368
									0.8166	0.1243	0.1204	0.8403	0.1362	0.1304	0.7944	0.1171	0.1153
		2	0.8444	0.1988	0.1882	0.7709	0.1843	0.1832	0.8223	0.1509	0.1465	0.8519	0.1674	0.1601	0.7948	0.1405	0.1389
									0.8163	0.1242	0.1217	0.8405	0.1362	0.1321	0.7936	0.1170	0.1163
		3	0.8422	0.2079	0.1955	0.7644	0.1941	0.1932	0.8218	0.1559	0.1505	0.8539	0.1740	0.1652	0.7923	0.1449	0.1430
									0.8151	0.1269	0.1238	0.8410	0.1397	0.1350	0.7911	0.1196	0.1187

**Table 3:** The APEs,  RMSEs and MRABs of the MLE, MPS and BEs for *R*(*x*) based on the PHT-ICS under various censoring schemes.

*T*	(*n,m*) Sch.		MLE			MPS			Baysian								
									SE			LINEX					
									Prior-1			Prior-1					
									Prior-2			Prior-2					
q→												–2			+2		
			**APE**	**RMSE**	**MRAB**	**APE**	**RMSE**	**MRAB**	**APE**	**RMSE**	**MRAB**	**APE**	**RMSE**	**MRAB**	**APE**	**RMSE**	**MRAB**
1.5	(40,20)	1	0.9829	0.0138	0.0107	0.9748	0.0198	0.0148	0.9787	0.0117	0.0087	0.9789	0.0115	0.0085	0.9785	0.0119	0.0088
									0.9798	0.0096	0.0071	0.9800	0.0094	0.0070	0.9796	0.0097	0.0072
		2	0.9831	0.0134	0.0108	0.9751	0.0192	0.0146	0.9785	0.0117	0.0087	0.9788	0.0115	0.0086	0.9783	0.0119	0.0089
									0.9795	0.0098	0.0073	0.9797	0.0097	0.0072	0.9793	0.0100	0.0074
		3	0.9818	0.0166	0.0125	0.9725	0.0241	0.0175	0.9781	0.0139	0.0099	0.9784	0.0136	0.0097	0.9778	0.0142	0.0101
									0.9793	0.0114	0.0082	0.9795	0.0112	0.0081	0.9791	0.0116	0.0083
1.5	(40,32)	1	0.9817	0.0140	0.0111	0.9735	0.0204	0.0157	0.9779	0.0122	0.0092	0.9782	0.0119	0.0090	0.9777	0.0124	0.0094
									0.9792	0.0098	0.0074	0.9794	0.0097	0.0073	0.9790	0.0100	0.0075
		2	0.9829	0.0140	0.0110	0.9749	0.0200	0.0149	0.9788	0.0119	0.0089	0.9790	0.0117	0.0088	0.9785	0.0122	0.0091
									0.9799	0.0097	0.0073	0.9800	0.0096	0.0072	0.9797	0.0099	0.0074
		3	0.9816	0.0144	0.0113	0.9727	0.0216	0.0164	0.9780	0.0121	0.0091	0.9783	0.0119	0.0089	0.9778	0.0124	0.0093
									0.9794	0.0097	0.0073	0.9795	0.0096	0.0072	0.9792	0.0099	0.0075
1.5	(80,40)	1	0.9834	0.0100	0.0082	0.9787	0.0129	0.0102	0.9808	0.0091	0.0072	0.9809	0.0090	0.0072	0.9807	0.0092	0.0073
									0.9812	0.0078	0.0062	0.9813	0.0077	0.0061	0.9811	0.0079	0.0062
		2	0.9833	0.0102	0.0082	0.9784	0.0131	0.0101	0.9804	0.0091	0.0070	0.9806	0.0090	0.0069	0.9803	0.0092	0.0071
									0.9809	0.0079	0.0060	0.9810	0.0078	0.0060	0.9808	0.0079	0.0061
		3	0.9815	0.0123	0.0095	0.9750	0.0170	0.0129	0.9789	0.0111	0.0083	0.9791	0.0110	0.0082	0.9788	0.0113	0.0084
									0.9797	0.0096	0.0072	0.9798	0.0094	0.0071	0.9796	0.0097	0.0072
1.5	(80,64)	1	0.9829	0.0100	0.0080	0.9782	0.0130	0.0100	0.9804	0.0091	0.0070	0.9805	0.0090	0.0070	0.9803	0.0092	0.0071
									0.9809	0.0078	0.0060	0.9810	0.0078	0.0060	0.9808	0.0079	0.0061
		2	0.9830	0.0099	0.0080	0.9782	0.0129	0.0100	0.9804	0.0091	0.0070	0.9805	0.0090	0.0069	0.9803	0.0092	0.0071
									0.9809	0.0078	0.0060	0.9810	0.0078	0.0060	0.9808	0.0079	0.0061
		3	0.9828	0.0109	0.0085	0.9775	0.0142	0.0108	0.9803	0.0098	0.0074	0.9804	0.0097	0.0074	0.9801	0.0099	0.0075
									0.9809	0.0084	0.0064	0.9810	0.0083	0.0063	0.9808	0.0085	0.0064
2.5	(40,20)	1	0.9836	0.0133	0.0104	0.9743	0.0201	0.0149	0.9790	0.0117	0.0087	0.9792	0.0115	0.0085	0.9787	0.0119	0.0088
									0.9798	0.0096	0.0071	0.9800	0.0095	0.0070	0.9797	0.0098	0.0072
		2	0.9826	0.0133	0.0105	0.9749	0.0190	0.0144	0.9784	0.0119	0.0089	0.9787	0.0117	0.0088	0.9782	0.0121	0.0091
									0.9795	0.0098	0.0073	0.9796	0.0097	0.0072	0.9793	0.0100	0.0075
		3	0.9805	0.0166	0.0125	0.9709	0.0249	0.0183	0.9771	0.0141	0.0104	0.9774	0.0138	0.0102	0.9768	0.0145	0.0107
									0.9785	0.0113	0.0084	0.9788	0.0111	0.0082	0.9783	0.0116	0.0085
2.5	(40,32)	1	0.9821	0.0135	0.0106	0.9745	0.0194	0.0146	0.9782	0.0122	0.0092	0.9785	0.0120	0.0091	0.9780	0.0125	0.0094
									0.9794	0.0099	0.0075	0.9796	0.0097	0.0074	0.9793	0.0101	0.0076
		2	0.9822	0.0141	0.0109	0.9746	0.0200	0.0148	0.9782	0.0126	0.0093	0.9784	0.0124	0.0091	0.9779	0.0129	0.0095
									0.9793	0.0103	0.0076	0.9794	0.0101	0.0075	0.9791	0.0105	0.0077
		3	0.9818	0.0144	0.0114	0.9737	0.0210	0.0158	0.9780	0.0126	0.0096	0.9783	0.0124	0.0094	0.9778	0.0129	0.0097
									0.9792	0.0102	0.0077	0.9794	0.0100	0.0076	0.9791	0.0104	0.0078
2.5	(80,40)	1	0.9837	0.0096	0.0079	0.9785	0.0128	0.0100	0.9810	0.0089	0.0071	0.9811	0.0089	0.0070	0.9809	0.0090	0.0072
									0.9814	0.0078	0.0062	0.9815	0.0077	0.0061	0.9813	0.0079	0.0062
		2	0.9830	0.0100	0.0080	0.9783	0.0129	0.0099	0.9803	0.0092	0.0071	0.9805	0.0091	0.0070	0.9802	0.0093	0.0072
									0.9808	0.0080	0.0061	0.9809	0.0079	0.0061	0.9807	0.0081	0.0062
		3	0.9825	0.0119	0.0095	0.9767	0.0162	0.0124	0.9799	0.0107	0.0083	0.9801	0.0106	0.0082	0.9798	0.0109	0.0084
									0.9806	0.0092	0.0071	0.9807	0.0090	0.0070	0.9804	0.0093	0.0072
2.5	(80,64)	1	0.9828	0.0097	0.0076	0.9784	0.0124	0.0095	0.9804	0.0092	0.0070	0.9805	0.0091	0.0070	0.9802	0.0093	0.0071
									0.9809	0.0080	0.0061	0.9810	0.0079	0.0061	0.9808	0.0081	0.0062
		2	0.9829	0.0098	0.0078	0.9784	0.0125	0.0096	0.9803	0.0092	0.0071	0.9805	0.0091	0.0070	0.9802	0.0093	0.0071
									0.9809	0.0080	0.0061	0.9810	0.0079	0.0061	0.9808	0.0081	0.0062
		3	0.9825	0.0105	0.0082	0.9775	0.0138	0.0105	0.9800	0.0098	0.0075	0.9801	0.0097	0.0074	0.9798	0.0099	0.0076
									0.9806	0.0084	0.0064	0.9807	0.0083	0.0064	0.9805	0.0085	0.0065

**Table 4:** The APEs, RMSEs and MRABs of the MLE, MPS and BEs for *H*(*x*) based on the PHT-ICS under various censoring schemes.

*T*	(*n,m*) Sch.		MLE			MPS			Baysian								
									SE			LINEX					
									Prior-1			Prior-1					
									Prior-2			Prior-2					
q→												–2			+2		
			**APE**	**RMSE**	**MRAB**	**APE**	**RMSE**	**MRAB**	**APE**	**RMSE**	**MRAB**	**APE**	**RMSE**	**MRAB**	**APE**	**RMSE**	**MRAB**
1.5	(40,20)	1	0.2011	0.1150	0.4296	0.2604	0.1326	0.4912	0.2301	0.0885	0.3168	0.2427	0.0952	0.3372	0.2189	0.0838	0.3035
									0.2302	0.0787	0.2789	0.2410	0.0846	0.2959	0.2205	0.0744	0.2684
		2	0.2027	0.1187	0.4461	0.2622	0.1359	0.4987	0.2333	0.0924	0.3285	0.2465	0.1003	0.3520	0.2217	0.0867	0.3129
									0.2338	0.0832	0.2938	0.2452	0.0902	0.3143	0.2237	0.0780	0.2796
		3	0.2058	0.1390	0.5082	0.2726	0.1637	0.5794	0.2313	0.1084	0.3798	0.2478	0.1201	0.4125	0.2171	0.1003	0.3586
									0.2315	0.0953	0.3329	0.2457	0.1050	0.3600	0.2193	0.0884	0.3146
1.5	(40,32)	1	0.2111	0.1153	0.4363	0.2697	0.1361	0.5111	0.2357	0.0894	0.3244	0.2484	0.0966	0.3459	0.2243	0.0842	0.3091
									0.2345	0.0789	0.2839	0.2454	0.0852	0.3024	0.2247	0.0742	0.2711
		2	0.2008	0.1172	0.4461	0.2593	0.1344	0.4975	0.2289	0.0900	0.3287	0.2414	0.0965	0.3481	0.2178	0.0854	0.3163
									0.2287	0.0795	0.2885	0.2394	0.0852	0.3053	0.2191	0.0755	0.2777
		3	0.2085	0.1157	0.4391	0.2715	0.1379	0.5110	0.2327	0.0893	0.3248	0.2464	0.0973	0.3486	0.2206	0.0837	0.3091
									0.2318	0.0788	0.2848	0.2436	0.0857	0.3050	0.2214	0.0739	0.2716
1.5	(80,40)	1	0.2057	0.0887	0.3355	0.2426	0.0968	0.3652	0.2215	0.0728	0.2720	0.2284	0.0752	0.2802	0.2150	0.0711	0.2664
									0.2221	0.0657	0.2444	0.2284	0.0680	0.2518	0.2163	0.0641	0.2394
		2	0.2082	0.0884	0.3290	0.2461	0.0966	0.3560	0.2251	0.0722	0.2638	0.2322	0.0751	0.2725	0.2185	0.0701	0.2579
									0.2262	0.0660	0.2399	0.2326	0.0687	0.2481	0.2202	0.0639	0.2341
		3	0.2210	0.1047	0.3801	0.2697	0.1232	0.4461	0.2343	0.0878	0.3114	0.2443	0.0937	0.3306	0.2251	0.0832	0.2978
									0.2342	0.0801	0.2831	0.2432	0.0855	0.3005	0.2260	0.0759	0.2707
1.5	(80,64)	1	0.2104	0.0865	0.3197	0.2478	0.0960	0.3525	0.2252	0.0722	0.2612	0.2322	0.0749	0.2701	0.2186	0.0701	0.2552
									0.2253	0.0655	0.2359	0.2315	0.0680	0.2443	0.2193	0.0635	0.2299
		2	0.2101	0.0866	0.3196	0.2476	0.0960	0.3524	0.2251	0.0722	0.2612	0.2321	0.0750	0.2702	0.2186	0.0702	0.2551
									0.2253	0.0656	0.2359	0.2316	0.0682	0.2444	0.2194	0.0637	0.2298
		3	0.2093	0.0930	0.3424	0.2499	0.1033	0.3786	0.2238	0.0774	0.2793	0.2315	0.0806	0.2901	0.2167	0.0751	0.2720
									0.2240	0.0704	0.2523	0.2309	0.0733	0.2615	0.2175	0.0682	0.2457
2.5	(40,20)	1	0.1997	0.1162	0.4355	0.2693	0.1380	0.5094	0.2316	0.0904	0.3234	0.2445	0.0973	0.3449	0.2202	0.0855	0.3093
									0.2321	0.0797	0.2829	0.2431	0.0858	0.3006	0.2222	0.0753	0.2713
		2	0.2075	0.1162	0.4336	0.2650	0.1343	0.4943	0.2342	0.0925	0.3294	0.2473	0.1003	0.3532	0.2226	0.0868	0.3134
									0.2339	0.0823	0.2909	0.2451	0.0892	0.3111	0.2238	0.0773	0.2771
		3	0.2192	0.1340	0.4931	0.2869	0.1643	0.5975	0.2408	0.1048	0.3708	0.2576	0.1168	0.4082	0.2262	0.0961	0.3452
									0.2386	0.0907	0.3196	0.2529	0.1008	0.3506	0.2262	0.0834	0.2987
2.5	(40,32)	1	0.2113	0.1150	0.4313	0.2676	0.1365	0.5035	0.2352	0.0921	0.3339	0.2480	0.0993	0.3563	0.2238	0.0869	0.3184
									0.2332	0.0802	0.2887	0.2440	0.0863	0.3071	0.2234	0.0758	0.2761
		2	0.2105	0.1191	0.4447	0.2670	0.1392	0.5082	0.2366	0.0944	0.3359	0.2495	0.1017	0.3594	0.2251	0.0889	0.3206
									0.2357	0.0826	0.2926	0.2467	0.0891	0.3119	0.2258	0.0778	0.2786
		3	0.2114	0.1208	0.4590	0.2716	0.1441	0.5346	0.2360	0.0943	0.3431	0.2499	0.1025	0.3682	0.2237	0.0883	0.3262
									0.2343	0.0821	0.2968	0.2461	0.0890	0.3172	0.2238	0.0770	0.2829
2.5	(80,40)	1	0.2054	0.0878	0.3313	0.2475	0.0980	0.3678	0.2220	0.0735	0.2741	0.2290	0.0760	0.2826	0.2154	0.0717	0.2678
									0.2227	0.0663	0.2464	0.2290	0.0686	0.2542	0.2168	0.0646	0.2407
		2	0.2114	0.0865	0.3219	0.2478	0.0957	0.3530	0.2266	0.0725	0.2655	0.2337	0.0755	0.2743	0.2200	0.0703	0.2587
									0.2269	0.0660	0.2404	0.2333	0.0687	0.2487	0.2209	0.0638	0.2344
		3	0.2114	0.1042	0.3909	0.2572	0.1197	0.4454	0.2265	0.0856	0.3168	0.2361	0.0904	0.3329	0.2177	0.0819	0.3052
									0.2267	0.0765	0.2819	0.2352	0.0808	0.2956	0.2188	0.0732	0.2719
2.5	(80,64)	1	0.2133	0.0851	0.3136	0.2489	0.0956	0.3487	0.2275	0.0733	0.2646	0.2345	0.0762	0.2748	0.2209	0.0710	0.2576
									0.2267	0.0662	0.2382	0.2330	0.0688	0.2474	0.2208	0.0641	0.2318
		2	0.2127	0.0854	0.3160	0.2489	0.0955	0.3487	0.2272	0.0731	0.2645	0.2342	0.0760	0.2744	0.2206	0.0708	0.2575
									0.2265	0.0660	0.2377	0.2329	0.0686	0.2467	0.2206	0.0640	0.2313
		3	0.2146	0.0904	0.3331	0.2538	0.1031	0.3761	0.2287	0.0768	0.2775	0.2366	0.0804	0.2895	0.2214	0.0741	0.2688
									0.2280	0.0691	0.2482	0.2351	0.0723	0.2589	0.2215	0.0666	0.2403

**Table 5:** ACLs and CPs of 95% intervals for θ and λ under MLE, MPS, and Bayesian HPD methods.

(n,m)	Sch.	ACI-NA (MLE)		ACI-NA (MPS)		HPD				ACI-NA (MLE)		ACI-NA (MPS)		HPD			
						Prior-1		Prior-2						Prior-1		Prior-2	
		ACL	CP	ACL	CP	ACL	CP	ACL	CP	ACL	CP	ACL	CP	ACL	CP	ACL	CP
		*T* = 1.5								*T* = 2.5							
		θ															
(40,20)	1	0.7199	94.50	0.6420	92.08	0.4862	96.60	0.4200	98.30	0.6145	96.50	0.5449	92.87	0.4428	96.60	0.3878	97.60
	2	0.9482	95.60	0.8286	93.90	0.5595	97.70	0.4691	98.80	0.6960	96.50	0.6227	94.19	0.4921	97.60	0.4271	98.50
	3	0.9827	94.50	0.8822	92.18	0.5768	97.90	0.4885	99.30	0.6475	94.40	0.5935	93.14	0.4834	97.10	0.4274	98.80
(40,32)	1	0.6776	94.90	0.6118	92.28	0.4742	96.80	0.4133	97.90	0.4568	95.30	0.4269	93.87	0.3759	95.90	0.3423	96.90
	2	0.7085	94.40	0.6357	92.38	0.4821	97.50	0.4181	98.80	0.5187	96.10	0.4794	94.38	0.4111	96.90	0.3685	97.90
	3	0.7391	92.70	0.6631	89.56	0.4996	95.90	0.4329	97.80	0.5166	94.60	0.4792	92.99	0.4123	96.30	0.3713	96.90
(80,40)	1	0.4520	95.20	0.4227	93.89	0.3744	96.20	0.3387	98.20	0.3730	96.00	0.3496	93.99	0.3233	96.20	0.2983	97.80
	2	0.5855	96.50	0.5357	94.38	0.4466	97.80	0.3923	98.60	0.4582	95.70	0.4272	94.38	0.3824	96.80	0.3468	97.90
	3	0.6088	95.80	0.5600	94.80	0.4687	97.20	0.4140	98.10	0.4440	95.70	0.4176	94.58	0.3771	96.60	0.3449	97.50
(80,64)	1	0.4439	94.50	0.4152	92.00	0.3702	95.80	0.3364	96.90	0.3124	95.20	0.2994	93.98	0.2820	95.90	0.2656	96.50
	2	0.4517	94.80	0.4219	92.10	0.3747	96.10	0.3400	96.90	0.3408	95.10	0.3244	93.69	0.3031	96.00	0.2830	96.60
	3	0.4880	94.80	0.4532	92.79	0.3974	96.50	0.3574	97.90	0.3468	95.40	0.3302	93.54	0.3084	96.20	0.2884	96.80
		λ															
(40,20)	1	1.2355	95.20	1.1183	90.48	0.8784	97.80	0.7419	98.40	1.1474	95.60	1.0307	90.16	0.8576	98.00	0.7309	98.80
	2	1.3303	96.90	1.2029	93.30	0.8943	99.10	0.7476	99.60	1.1842	96.00	1.0786	91.88	0.8638	98.90	0.7338	99.20
	3	1.4569	94.50	1.2713	87.37	0.9458	98.50	0.7833	99.40	1.2407	94.10	1.0825	87.59	0.8980	97.90	0.7594	99.30
(40,32)	1	1.2052	94.80	1.0915	89.27	0.8652	97.80	0.7331	99.00	1.0627	95.80	0.9708	90.95	0.8260	98.20	0.7161	99.00
	2	1.2491	95.20	1.1296	90.98	0.8815	98.00	0.7459	98.90	1.0889	94.30	0.9951	90.37	0.8353	97.80	0.7201	99.10
	3	1.2750	94.40	1.1338	88.25	0.8881	98.10	0.7492	99.00	1.1275	94.40	1.0147	88.38	0.8540	98.10	0.7335	99.40
(80,40)	1	0.8295	95.30	0.7787	90.98	0.6903	97.00	0.6168	98.50	0.7670	94.80	0.7182	91.29	0.6587	96.90	0.5971	98.20
	2	0.8861	95.30	0.8299	93.57	0.7118	98.00	0.6269	99.00	0.8001	95.60	0.7542	91.87	0.6741	97.10	0.6050	98.40
	3	0.9685	95.90	0.8701	89.70	0.7601	98.10	0.6632	98.90	0.8918	94.70	0.8081	89.36	0.7355	97.30	0.6521	98.40
(80,64)	1	0.8169	95.70	0.7665	91.70	0.6829	97.90	0.6109	98.70	0.7201	95.60	0.6803	92.37	0.6309	96.60	0.5777	97.60
	2	0.8207	95.50	0.7700	92.00	0.6844	97.90	0.6123	98.80	0.7385	95.60	0.6967	92.28	0.6415	97.10	0.5845	97.90
	3	0.8795	95.50	0.8134	90.79	0.7178	97.70	0.6350	98.40	0.7713	95.70	0.7197	91.01	0.6634	97.20	0.6011	98.10

**Table 6:** ACLs and CPs of 95% intervals for *R*(*x*) and *H*(*x*) under MLE, MPS, and Bayesian HPD methods.

(n,m)	Sch.	ACI-NA (MLE)		ACI-NA (MPS)		HPD				ACI-NA (MLE)		ACI-NA (MPS)		HPD			
						Prior-1		Prior-2						Prior-1		Prior-2	
		ACL	CP	ACL	CP	ACL	CP	ACL	CP	ACL	CP	ACL	CP	ACL	CP	ACL	CP
		*T* = 1.5								*T* = 2.5							
		R(x)															
(40,20)	1	0.0438	82.00	0.0611	90.88	0.0480	98.20	0.0425	99.20	0.0418	81.30	0.0617	91.57	0.0474	97.80	0.0425	99.10
	2	0.0437	82.70	0.0613	90.90	0.0484	98.50	0.0430	99.40	0.0442	83.10	0.0610	92.28	0.0484	98.00	0.0430	99.10
	3	0.0488	80.90	0.0682	89.08	0.0518	97.90	0.0459	98.50	0.0515	83.80	0.0711	91.32	0.0538	97.70	0.0474	99.30
(40,32)	1	0.0463	83.40	0.0636	91.47	0.0493	98.40	0.0433	99.10	0.0448	84.60	0.0606	90.95	0.0485	97.30	0.0430	99.20
	2	0.0438	81.60	0.0608	90.28	0.0479	98.40	0.0423	99.10	0.0445	83.20	0.0603	90.77	0.0486	97.80	0.0432	99.10
	3	0.0477	84.60	0.0666	92.77	0.0502	98.50	0.0442	99.30	0.0464	83.20	0.0636	91.38	0.0500	98.20	0.0443	98.90
(80,40)	1	0.0357	84.70	0.0439	91.48	0.0362	95.80	0.0334	97.20	0.0346	85.10	0.0439	92.59	0.0355	95.60	0.0331	96.70
	2	0.0362	86.00	0.0449	93.47	0.0368	96.90	0.0339	98.50	0.0362	87.40	0.0444	93.98	0.0366	97.10	0.0338	98.00
	3	0.0428	88.60	0.0542	93.50	0.0426	96.80	0.0389	97.80	0.0401	84.00	0.0506	91.27	0.0408	94.90	0.0376	96.80
(80,64)	1	0.0365	87.90	0.0450	92.40	0.0367	96.80	0.0338	97.40	0.0359	88.50	0.0435	93.27	0.0362	95.10	0.0337	97.10
	2	0.0365	87.80	0.0449	92.60	0.0367	96.40	0.0338	97.60	0.0360	88.50	0.0439	92.69	0.0364	96.10	0.0337	97.10
	3	0.0379	87.80	0.0473	91.79	0.0382	96.40	0.0350	97.10	0.0379	88.00	0.0466	93.43	0.0383	96.30	0.0353	97.40
		H(x)															
(40,20)	1	0.4069	86.20	0.4774	91.28	0.3881	97.00	0.3651	97.70	0.4058	85.00	0.4913	91.47	0.3919	96.40	0.3683	97.80
	2	0.4141	86.50	0.4851	92.80	0.3943	96.90	0.3722	97.50	0.4175	87.00	0.4852	92.48	0.3939	96.60	0.3713	97.90
	3	0.4467	84.30	0.5298	89.98	0.4256	95.10	0.4021	96.60	0.4664	87.00	0.5431	91.73	0.4358	97.10	0.4082	98.40
(40,32)	1	0.4185	87.40	0.4841	91.88	0.3915	97.40	0.3677	97.80	0.4168	87.40	0.4817	90.95	0.3914	96.70	0.3661	98.20
	2	0.4055	86.10	0.4754	91.48	0.3858	97.70	0.3630	98.30	0.4151	86.50	0.4812	91.17	0.3938	97.10	0.3695	98.30
	3	0.4291	88.70	0.5005	92.87	0.4018	97.10	0.3779	98.50	0.4293	86.40	0.4986	91.18	0.4053	97.50	0.3793	98.50
(80,40)	1	0.3279	88.60	0.3566	92.28	0.3019	95.70	0.2883	96.70	0.3275	88.70	0.3614	92.09	0.3028	95.00	0.2897	96.90
	2	0.3346	90.30	0.3633	93.47	0.3063	95.30	0.2928	97.40	0.3341	90.80	0.3616	93.67	0.3064	95.90	0.2931	97.10
	3	0.3941	90.50	0.4309	92.20	0.3555	95.20	0.3387	96.90	0.3788	87.30	0.4156	91.27	0.3468	94.70	0.3307	96.20
(80,64)	1	0.3336	89.80	0.3615	93.10	0.3044	95.40	0.2907	97.00	0.3329	90.30	0.3603	92.57	0.3057	94.80	0.2917	96.70
	2	0.3338	89.90	0.3618	93.10	0.3047	95.50	0.2908	97.10	0.3332	90.30	0.3608	92.99	0.3059	95.30	0.2913	97.10
	3	0.3476	89.40	0.3789	92.39	0.3166	94.60	0.3018	95.80	0.3519	90.00	0.3819	93.03	0.3211	95.50	0.3054	96.80

From Tables 1–6, in terms of the lowest RMSE, MRAB, and ACL values as well as the highest CP values, the following observations are drawn:

- All suggested point/interval estimates of θ, λ, *R*(*x*) and *H*(*x*) have better performances; it is our general note.
- Among point estimators, Bayes-Linex (*q* =  + 2) consistently achieves the lowest RMSE and MRAB, followed by Bayes-SE, Bayes-Linex (*q* = −2), with MPS either outperforming or matching MLE.
- As *n* or *m* increases-or as $\sum_{i=1}^{m}R_{i}$ decreases-estimates of θ, λ, *R*(*x*), and *H*(*x*) improve markedly, confirming the consistency of the proposed methods.
- As *T* increases, in most cases, the simulated RMSE, MRAB, and ACL values of θ, λ, *R*(*x*), and *H*(*x*) decrease while their CPs increase.
- While no single scheme consistently dominates across all criteria, Scheme-1 frequently provides more accurate estimates in a broad range of scenarios.
- Comparing the point estimation methods of θ, λ, *R*(*x*) or *H*(*x*), in terms of the lowest RMSEs and MRABs, the Bayes MCMC estimates performed better against the LINEX loss than the SE loss and both are more favorable compared to the classical estimates. It is an anticipated result due to the Bayes MCMC estimates having included additional prior information.
- Interval estimation hierarchy: HPD < ACI-NA, with MPS-based ACI-NA yielding shorter ACLs than MLE-based; overall, Bayesian intervals (HPD) outperform frequentist ones thanks to the gamma prior.
- Comparing the priors 1 and 2, the Bayesian (point/interval) estimates using Prior-2 provide better results than Prior-1 for all unknown parameters. This result is due to the fact that the variance of Prior-2 is lower than the variance of Prior-1.
- As a result, the Bayes MCMC estimation method is recommended to estimate the parameters or its reliability characteristics of the inverted exponentiated Rayleigh distribution in the presence of data obtained from PHT-ICS.

## 6 Real data analysis

To illustrate the adaptability and usefulness of the proposed estimation methodologies to a real-life phenomenon, in this section, we shall present the analysis of a data set taken from the medicine area. This data, reported by Wingo [55] and reanalyzed by Soliman et al. [56], represents a relief time (in hours) for fifty arthritic patients receiving a fixed dosage of this medication. In Table 7, for computational convenience, each relief time point is divided by ten.

**Table 7:** Relief times for arthritic patients.

2.9	2.9	3.4	3.4	3.5	3.6	3.6	3.6	4.4	4.4
4.6	4.6	4.9	4.9	5.0	5.0	5.2	5.4	5.5	5.5
5.5	5.6	5.7	5.8	5.9	5.9	6.0	6.0	6.1	6.1
6.2	6.4	6.8	7.0	7.0	7.1	7.1	7.1	7.2	7.3
7.5	7.5	8.0	8.0	8.1	8.2	8.4	8.4	8.4	8.7

Before proceeding further, we need to check the validity of the IER distribution for arthritic patients data. Therefore, the Kolmogorov–Smirnov (KS) statistic (along its *P*-value) is calculated. For this purpose, using Table 7, the MLEs $\hat{\theta }$ and $\hat{\lambda }$ of θ and λ, respectively, must be calculated first. However, the MLEs (along their standard errors (SEs)) of θ and λ are 55.334(8.3398) and 3.8129(0.9978), respectively, while the K-S(*P*-value) is 0.127(0.394). It shows that the IER distribution fits the arthritic patients data quite well.

**Fig 3:**
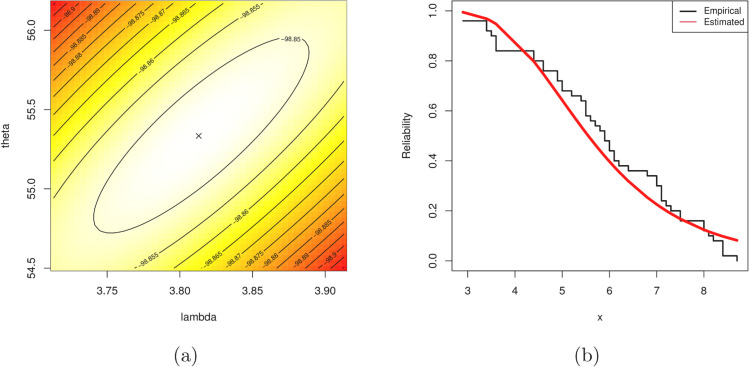
(a) Contour plot of the log-likelihood function of θ and λ, and (b) estimated/empirical reliability function of the IER distribution from arthritic patients data.

To examine the existence and uniqueness of the computed MLEs $\hat{\theta }$ and $\hat{\lambda }$, based on the arthritic patients data set, the contour plot of the joint log-likelihood function for various choices of θ and λ is plotted and depicted in Fig 3(a). It shows that the MLEs $\hat{\theta }$ and $\hat{\lambda }$ exist and are unique. It is also evident, to carried out any upcoming evaluations, that the most useful starting values ofθ and λ are close to 55.334 and 3.8129, respectively. Additionally, the plot of estimated/empirical reliability function of the IER lifetime model is displayed in Fig 3(b). It also supports the numerical goodness-of-fit findings.

From the complete arthritic patients data, by taking *m* = 25 with different choices of *R* and *T*, four different artificial PHT-ICS are generated and reported in Table 8. For brevity, R=(1,0,0,1) is used as $\text{R}=(1,0^{2},1)$.

**Table 8:** PHT-ICS from arthritic patients data.

Scheme	Sample	*T*(*D*)	$R_{\omega }^{*}$	Censored Data
${𝒮}_{1}$	(1^25^)	7.4(20)	10	2.9, 3.4, 3.5, 3.6, 4.4, 4.6, 4.9, 5.0, 5.2, 5.5, 5.5, 5.7, 5.9, 6.0, 6.1, 6.2, 6.8, 7.0, 7.1, 7.2
${𝒮}_{2}$	(25,0^24^)	8.5(25)	0	2.9, 3.4, 3.5, 3.6, 4.4, 4.6, 4.9, 5.0, 5.5, 5.7, 5.8, 5.9, 6.0, 6.0, 6.1, 6.2, 6.4, 6.8, 7.1, 7.2, 7.3, 7.5, 7.5, 8.0, 8.1
${𝒮}_{3}$	(0^12^,25,0^12^)	6.9(21)	4	2.9, 2.9, 3.4, 3.4, 3.5, 3.6, 3.6, 3.6, 4.4, 4.4, 4.6, 4.6, 4.9, 4.9, 5.0, 5.4, 5.5, 5.5, 5.7, 6.1, 6.8
${𝒮}_{4}$	(0^24^,25)	6.1(25)	0	2.9, 2.9, 3.4, 3.4, 3.5, 3.6, 3.6, 3.6, 4.4, 4.4, 4.6, 4.6, 4.9, 4.9, 5.0, 5.0, 5.2, 5.4, 5.5, 5.5, 5.5, 5.6, 5.7, 5.8, 5.9

For each censored sample presented in Table 8, the maximum likelihood estimates (together with their standard errors) and the corresponding 95% ACI-NA/ACI-NL estimates (with their interval lengths) of θ, λ, *R*(*x*), and *H*(*x*) are calculated. Due to the absence of prior information regarding the unknown IER parameters θ and λ from the dataset of arthritic patients, the Bayes estimates (together with their standard errors) and the HPD interval estimates (with their interval lengths) are assessed using inappropriate gamma priors. In numerical logic, all unspecified hyperparameters *a*, *b*, *c*, and *d* are assigned a value of 0.001. The MCMC sampler is executed 50,000 times, with the initial 10,000 iterations disregarded as burn-in, to derive the Bayesian point and interval estimates. Tables 9 and 10 exhibit the computed point and interval estimates of θ, λ, *R*(*x*), and *H*(*x*) (at *t* = 5), respectively. Table 9 clearly indicates that the MCMC estimates of θ, λ, *R*(*x*), and *H*(*x*) exhibit superior performance for the minimal standard errors and interval lengths. The point (or interval) estimates derived from the maximum likelihood and Bayesian estimation approaches are notably similar.

**Table 9:** Average estimates with their SEs of θ, λ, *R*(*x*) and *H*(*x*) from arthritic patients data.

Sample	Par.	MLE		SE		LINEX					
q→						–3		–0.03		+3	
${𝒮}_{1}$	θ	46.565	9.6687	46.463	0.1417	46.478	0.0864	46.463	0.1010	46.449	0.1160
	λ	1.3097	0.4772	1.2119	0.1357	1.2252	0.0845	1.2121	0.0976	1.1986	0.1111
	*R*(5)	0.8017	0.0461	0.8144	0.0182	0.8147	0.0130	0.8144	0.0127	0.8142	0.0124
	*H*(5)	0.1794	0.0407	0.1664	0.0183	0.1667	0.0127	0.1664	0.0129	0.1662	0.0132
${𝒮}_{2}$	θ	60.223	9.3647	60.122	0.1407	60.136	0.0862	60.122	0.1005	60.107	0.1152
	λ	4.5942	1.4493	4.4914	0.1432	4.5063	0.0880	4.4916	0.1027	4.4764	0.1178
	*R*(5)	0.6487	0.0699	0.6538	0.0081	0.6539	0.0052	0.6538	0.0052	0.6538	0.0051
	*H*(5)	0.4374	0.0875	0.4287	0.0129	0.4289	0.0085	0.4287	0.0086	0.4286	0.0087
${𝒮}_{3}$	θ	41.240	6.9008	41.139	0.1408	41.153	0.0865	41.139	0.1008	41.124	0.1154
	λ	1.9698	0.7071	1.8679	0.1416	1.8823	0.0875	1.8680	0.1018	1.8532	0.1165
	*R*(5)	0.6569	0.0606	0.6703	0.0195	0.6706	0.0137	0.6703	0.0134	0.6700	0.0131
	*H*(5)	0.3091	0.0756	0.2939	0.0217	0.2942	0.0149	0.2939	0.0152	0.2935	0.0156
${𝒮}_{4}$	θ	39.427	6.7324	39.326	0.1412	39.341	0.0863	39.326	0.1008	39.312	0.1156
	λ	1.7280	0.5543	1.6293	0.1371	1.6428	0.0851	1.6294	0.0986	1.6157	0.1123
	*R*(5)	0.6704	0.0554	0.6849	0.0210	0.6852	0.0148	0.6849	0.0145	0.6846	0.0141
	*H*(5)	0.2838	0.0613	0.2683	0.0221	0.2686	0.0152	0.2683	0.0155	0.2679	0.0159

**Table 10:** Interval estimates of θ, λ, *R*(*x*) and *H*(*x*) from arthritic patients data.

Sample	Par.	ACI-NA			BCI		
		ACI-NL			HPD		
		Lower	Upper	IL	Lower	Upper	IL
${𝒮}_{1}$	θ	27.614	65.515	37.901	46.271	46.659	0.3879
		30.996	69.952	38.955	46.278	46.664	0.3866
	λ	0.3744	2.2450	1.8706	1.0276	1.3958	0.3682
		0.6412	2.6750	2.0337	1.0358	1.4000	0.3642
	*R*(5)	0.7113	0.8921	0.1808	0.7893	0.8401	0.0508
		0.7162	0.8974	0.1812	0.7903	0.8407	0.0504
	*H*(5)	0.0997	0.2590	0.1594	0.1411	0.1916	0.0505
		0.1150	0.2797	0.1647	0.1407	0.1907	0.0501
${𝒮}_{2}$	θ	41.868	78.577	36.709	59.929	60.317	0.3883
		44.401	81.681	37.280	59.937	60.324	0.3866
	λ	1.7536	7.4349	5.6813	4.2933	4.6836	0.3903
		2.4756	8.5259	6.0502	4.2981	4.6873	0.3892
	*R*(5)	0.5116	0.7857	0.2741	0.6417	0.6664	0.0247
		0.5251	0.8013	0.2762	0.6411	0.6655	0.0244
	*H*(5)	0.2658	0.6089	0.3432	0.4099	0.4473	0.0374
		0.2954	0.6475	0.3521	0.4099	0.4472	0.0373
${𝒮}_{3}$	θ	27.714	54.765	27.051	40.946	41.334	0.3875
		29.708	57.246	27.538	40.954	41.339	0.3855
	λ	0.5839	3.3556	2.7717	1.6699	2.0572	0.3873
		0.9747	3.9808	3.0061	1.6808	2.0639	0.3831
	*R*(5)	0.5382	0.7756	0.2374	0.6433	0.6988	0.0555
		0.5483	0.7870	0.2387	0.6430	0.6982	0.0551
	*H*(5)	0.1609	0.4573	0.2964	0.2629	0.3238	0.0610
		0.1914	0.4993	0.3079	0.2650	0.3253	0.0603
${𝒮}_{4}$	θ	26.232	52.622	26.390	39.134	39.522	0.3878
		28.213	55.099	26.886	39.141	39.528	0.3860
	λ	0.6415	2.8145	2.1730	1.4434	1.8153	0.3719
		0.9215	3.2404	2.3190	1.4528	1.8228	0.3700
	*R*(5)	0.5619	0.7789	0.2170	0.6556	0.7148	0.0592
		0.5702	0.7882	0.2180	0.6554	0.7143	0.0589
	*H*(5)	0.1636	0.4040	0.2405	0.2378	0.2990	0.0612
		0.1858	0.4335	0.2477	0.2375	0.2982	0.0607

**Fig 4:**
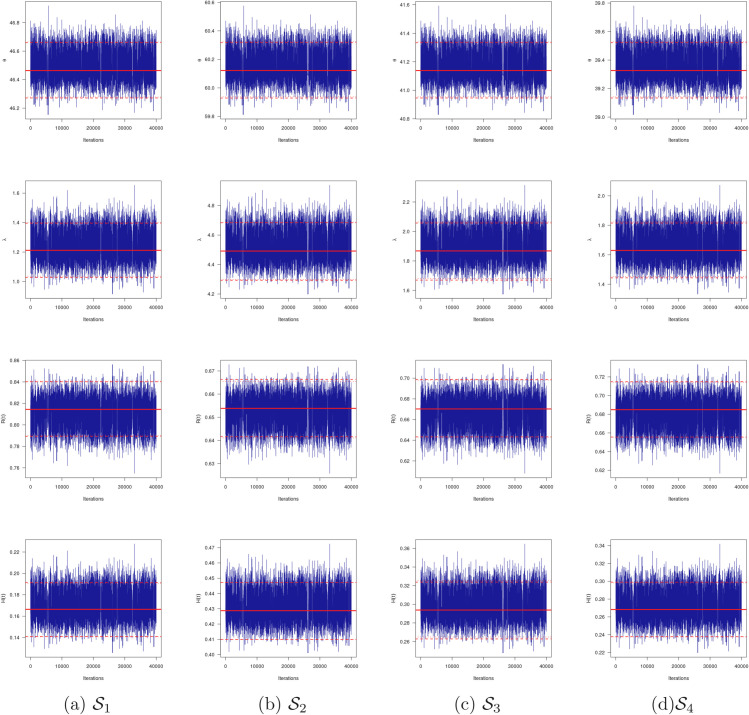
Trace plots of θ, λ, R(x) and H(x) from arthritic patients data.

**Fig 5:**
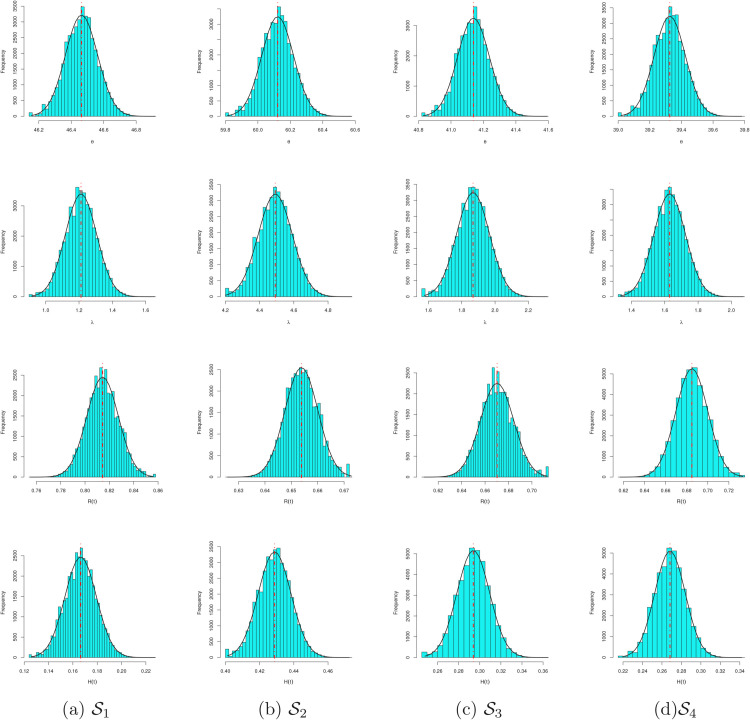
Histogram and kernel density estimates of θ, λ, R(x) and H(x) from arthritic patients data.

To assess the convergence of MCMC procedure, trace plots based on 40,000 MCMC values of θ, λ, *R*(*x*) and *H*(*x*) are plotted in Fig 4. Furthermore, based on the same 40,000 MCMC values, the marginal PDFs with their histograms using the Gaussian kernel of θ, λ, *R*(*x*) and *H*(*x*) are plotted in Fig 4. For distinguish, in each trace plot, the sample mean (has soled (-) line), two bounds of 95% BCI (has dashed (- - -) line) and two bounds of 95% HPD interval (has dotted (⋯) line). Also, in each histogram plot, the sample mean is referred by vertical dotted-dashed line. It is clear that the MCMC algorithm converges satisfactorily and shows that ignoring the first 10,000 samples is an appropriate size to erase the effect of the initial guesses. It is observed, from Fig 5, that the generated posteriors of θ, λ, *R*(*x*) and *H*(*x*) for ${𝒮}_{i},\;i=1,2,3,4$ are fairly-symmetrical. In addition, for all generated samples listed in Table 8, different useful statistics for MCMC draws of θ, λ, *R*(*x*) and *H*(*x*) after bun-in, namely: mean, mode, quartiles ($Q_{1},Q_{2},Q_{3}$), standard deviation (SD) and skewness are also obtained and reported in Table 11. It supports the same findings reported in Tables 9 and 10.

**Table 11:** Vital statistics for MCMC outputs of θ, λ, *R*(*x*) and *H*(*x*) from arthritic patients data.

Sample	Par.	Mean	Mode	$Q_{1}$	$Q_{2}$	$Q_{3}$	SD	Skewness
${𝒮}_{1}$	θ	46.463	46.316	46.395	46.465	46.528	0.0992	–0.0038
	λ	1.2119	1.0533	1.1483	1.2122	1.2745	0.0941	–0.0043
	*R*(5)	0.8144	0.8355	0.8057	0.8142	0.8230	0.0130	0.0523
	*H*(5)	0.1664	0.1452	0.1577	0.1665	0.1750	0.0129	–0.0043
${𝒮}_{2}$	θ	60.122	60.097	60.053	60.124	60.185	0.0983	0.0074
	λ	4.4914	4.2000	4.4258	4.4929	4.5579	0.0997	–0.0491
	*R*(5)	0.6538	0.6718	0.6495	0.6537	0.6580	0.0063	0.0640
	*H*(5)	0.4287	0.4012	0.4224	0.4287	0.4352	0.0096	–0.0428
${𝒮}_{3}$	θ	41.139	41.113	41.070	41.140	41.202	0.0982	0.0071
	λ	1.8679	1.5755	1.8042	1.8696	1.9334	0.0983	–0.0768
	*R*(5)	0.6703	0.7132	0.6607	0.6699	0.6794	0.0142	0.1389
	*H*(5)	0.2939	0.2480	0.2838	0.2942	0.3042	0.0155	–0.0754
${𝒮}_{4}$	θ	39.326	39.179	39.258	39.328	39.391	0.0987	-0.0014
	λ	1.6293	1.4716	1.5653	1.6296	1.6925	0.0951	0.0012
	*R*(5)	0.6849	0.7087	0.6746	0.6847	0.6949	0.0152	0.0646
	*H*(5)	0.2683	0.2432	0.2578	0.2684	0.2787	0.0157	0.0012

Finally, based on the arthritic patients data, we can draw the decision that the proposed estimation methodologies provide a good explanation of the IER lifetime model when a sample generated from the PHT-ICS.

## 7 Concluding remarks

This study investigates the utilization of ML, MPS and Bayesian inference in the IER distribution through a PHT-ICS. Numerical methods are employed to obtain the ML and MPS estimates of the unknown parameters, reliability and hazard rate function. Utilizing the asymptotic properties of the ML estimates, we derive the approximate confidence intervals for the unknown parameters, as well as the reliability and hazard rate functions. Alternatively, we evaluate Bayesian estimation using two loss functions: the squared error and LINEX loss functions. We obtain estimates with the MCMC methodology. Concurrently, we compute the Bayes credible intervals with the highest posterior density for the different unknown parameters. We did simulation study to evaluate the efficacy of various estimations. This study’s simulation part computes the average root mean square errors and mean relative absolute biases to evaluate the precision of the point estimations. The average interval lengths and coverage probability are examined to assess the dependability of the interval estimates. The simulation results indicate that Bayesian estimation, with informative priors, is more credible than ML and MPS estimates across all scenarios. The Bayesian estimates employing the LINEX loss function surpass all alternative estimates. Moreover, the highest posterior density Bayes credible intervals demonstrate the most concise average interval lengths and superior coverage probability relative to the approximation confidence intervals. The genuine dataset was acquired from the medical domain, specifically from a cohort of fifty arthritis patients who received a uniform dosage of this medication. The data was studied to demonstrate the viability of the several methodologies assessed. Future study may necessitate the expansion of the proposed approaches to include the competing risk model or accelerated life testing. Future research may concentrate on, among other aspects, (1) investigating the estimation of entropy measures within a competing risks model; (2) addressing estimation challenges of entropy measures in accelerated life tests; and (3) exploring alternative estimation techniques beyond maximum likelihood, including weighted least squares, and least squares.
